# Urinary Diseases and Their Treatment

**Published:** 1839-04-01

**Authors:** 


### Medico-Chirurgical Review,

No. LX.

[No. 20 of a Decennial Series.]

JANUARY 1, TO APRIL 1, 1839.

### ON DISEASES OF THE URINARY ORGANS.

I. URINARY DISEASES AND THEIR TREATMENT. By *Robert Willis*, M.D., Licentiate of the Royal College of Physicians, Physician to the Royal Infirmary for Children, &c. &c.

II. ON GRANULAR DEGENERATION OF THE KIDNIES, AND ITS CONNEXION WITH DROPSY, INFLAMMATIONS, AND OTHER DISEASES. By *Robert Christison*, M.D. F.R.S.E. &c. &c. Octavo, pp. 288. Edinb. 1839.

III. TRAITÉ DES MALADIES DES REINS, ET DES ALTÉRATIONS DE LA SÉCRÉTION URINAIRE, ÉTUDIÉES EN ELLE-MEMES, ET DANS LEURS RAPPORTS AVEC LES MALADIES DES URETERES, DE LA VESSIE, DE LA PROSTATE, DE L'URETHRE, &c. Avec un Atlas in folio. Par *P. Rayer*, Médecin de l'Hôpital de la Charité, &c. &c. Octavo. Tome Premier, pp. 638. Six Planches gravèes. A Paris, chez J. B. Bailliere.

THE scope and object of the work of Dr. Willis must be, from our former notice of it, tolerably familiar to our readers.

The title of that of Dr. Christison explains its more limited design. It is solely occupied with the "granular degeneration" of the kidnies, and is intended to illustrate, to amplify, perhaps correct, the views of Dr. Bright. Its author's anticipations, as well as his confidence, may be judged from the following copious passage in his Preface:—

"The apathy with which the invaluable discoveries of *Dr. Bright* continue to be regarded by many is most unaccountable. It is only within the last two years that medical men in this city have generally admitted the accuracy of his researches. I still meet with respectable members of the profession who seem inclined to adhere to their scepticism. And in a late visit to London I found to my surprise that by some medical gentlemen of the metropolis the doctrines of Dr. *Bright* continue to be called in question. This is surely a very strange result of the numerous extended inquiries which have now been made, and all with one invariable result. I do not know a single investigation, of such extent as to deserve the name, which has contradicted in any material point the general principles first announced in 1827. And as to confirmatory researches,

I shall take the liberty of expressing my sentiments in the language of *Dr. Osborne,* who remarks, that 'the number of observations recorded' (as this be it observed so far back as 1834) 'must be admitted to have been greater than has within many years been brought to bear on any one individual proposition in medical science.'

Were an apology wanted then for the appearance of the present treatise, the preceding exposition might perhaps be considered not inadequate. But other reasons have also actuated me. The subject I have undertaken to elucidate has been more or less an express object of study with me ever since the publication of Dr. Bright's Reports in 1827. Some of the general facts which have thus been deduced have gone forth in an imperfect form through the medium of my lectures delivered from time to time in the Clinical Courses of this University during the last six years. Occasionally I perceive from the Journals, that others have been expending much pains in ascertaining points which have here been for some time determined; and that others call in question facts which have been long placed out of the reach of controversy. A few received doctrines, of much importance both to pathology and to practice, have appeared to me doubtful, if not inconsistent with facts, and therefore to deserve early revision. One interesting department, the pathological condition of the blood, has hitherto been barely touched upon, and that chiefly in points which were started in my paper in 1829. On farther investigation it has appeared to me, not only that the special facts formerly advanced in an isolated shape may be now stated with full confidence in more general terms, but likewise that the entire subject may be presented with new features of interest. Lastly, I had long thought that the students of this University and the profession at large stood in need of an easily accessible work on Granular Degeneration of the Kidnies. Nor has the publication of the excellent memoir of *Dr. Osborne* altered this opinion; since the ground we have taken up is somewhat different, and the doctrines to which we have been conducted are not always in accord.

It is necessary to explain, that this treatise was framed in the first instance exclusively from the results of personal experience, based on the earliest work of *Dr. Bright.* Its substance indeed formed part of my Clinical Lectures delivered in 1831, as well as frequently since; and it was first written without reference to any researches published subsequently to those of *Dr. Gregory,* which made their appearance during the same year. Some valuable obervations have since been added from the publications of later authors. But these authors may also recognize apparently some of their facts unacknowledged; for I did not think it necessary to refer to others what had long been previously known to myself, and repeatedly made public in the discharge of my professional duties." xiii.

It is clear that Dr. Christison's observations must be possessed of high importance, for, he himself assures us, that they have determined points on which others are labouring in vain—that they place beyond the reach of controversy much that is controverted—that they render it probable that a few received doctrines ought to be received no longer—and that they establish some general conclusions of no mean consequence in reference to the pathological condition of the blood. All very excellent things, and quite enough to apologise for the publication of a book.

M. Rayer explains, in a preface of some pages, the object of *his* labours. He admits, what is undeniable, that the diseases of the kidney, and, particularly, the alterations of the urine, have hitherto received little attention in France. There cannot indeed be a more pregnant instance of the absence of a practical turn in our neighbours, than their preference of morbid anatomy to pathology. In the latter we have commenced and carried on investigations to which the continental physicians have been quite indifferent —while, in the former, we need not say how far they have outstripped us.

It would be idle to descant to the English reader on the value of the study of the alterations of the urine. It is necessary to awake the attention of his countrymen, and therefore M. Rayer may be excused for dwelling on it.

M. Rayer has studied and describes the alterations of the urine as they depend on excess or defect of some of its natural constituents—or as they result from the accidental presence of other organic principles, proceeding from the blood or from foreign matters accidentally introduced into the economy by absorption.

M. Rayer acknowledges the value of the ordinary tests employed to determine the condition of the urine. But he also calls attention to the employment of the microscope, and strongly insists on its utility. By its means, we promptly determine the nature of the deposits of the urine, or of substances suspended in it, and arrive at an acquaintance with organic matters, mucus, fragments of epithelium, small quantities of pus or blood, spermatic animalcules, substances or objects, which, without the microscope, present insurmountable difficulties to the inquirer.

The dominant idea of M. Rayer, that which has presided over his investigations, and tinges all his conclusions, is, that no diseases are absolutely local, but that all have a tendency to dissemination, and to implication of the system. We need scarcely observe that this opinion has long formed the basis of practice in England, at all events, since the publication of the work of Mr. Abernethy. Here then, as elsewhere, we may justly claim for our country and our countrymen that merit which is fairly due to them—the merit of being in advance of our continental brethren, in most that relates to the practical application of medicine.

We shall now return to the work of Dr. Willis. We left him on the brink of his fourth chapter, and we shall now enter on it. That chapter is intituled :—

I.—MORBID STATES IN WHICH THE URINE CONTAINS IN SOLUTION OR AS PRECIPITATES CERTAIN PRINCIPLES WHICH DO NOT OCCUR IN THE HEALTHY SECRETION, BUT APPEAR TO BE DERIVED IMMEDIATELY FROM ONE OR OTHER OF THESE.*

Dr. Willis remarks, that one of the many interesting discoveries of modern chemistry, is the convertibility of some proximate animal principles into one another—nay, the artificial formation of some from their ultimate elements. Urea, for example, is said to have been formed by Wœhler.

"If we set out," says Dr. W. "from this peculiar organic principle, the ultimate composition of which appears to be 46.65 nitrogen, 19.97 carbon, 6.65 hydrogen, and 26.65 oxygen, ($H^2$ $C^2$ $H^4$ $O^2$), and suppose the proportions of nitrogen and hydrogen to be lessened, whilst those of carbon and oxygen are increased, we shall have lithic or uric acid produced,—33.37 nitrogen, 36.00

* It is difficult to understand the construction of this sentence.

carbon, 2,36 hydrogen, 28,27 oxygen ($N^4 C^5 H^4 O^3$.) Again, if we suppose the nitrogen, carbon, and hydrogen, to remain as in lithic acid, but the quantity of oxygen to be diminished to the extent of one atom, we shall have the lithic or uric oxide (xanthic oxide Marcet,) produced—($N^4 C^5 H^4 O^2$). Farther, if the nitrogen and carbon fall short, whilst the hydrogen and oxygen increase in relative quantity, we have another element of morbid urine formed, namely, cystine, (cystic oxide, Wollaston,) as appears by the composition of this substance, 11.85 nitrogen, 29.88 carbon, 5.12 hydrogen, 53.15 oxygen, ($N C^3 H^6 O^4$). Another very remarkable quaternary compound, occasionally met with in morbid urine, is carbonate of ammonia, for the presence of which we should, without the lights afforded us by chemistry, be greatly at a loss to account. But when we know that the elementary composition of urea is identical with that of the cyanate of ammonia; that if one atom of this substance and one atom of water come to decompose each other, which readily happens, and that the product is exactly one atom of *carbonate of ammonia*, we have no difficulty whatever in explaining the occurrence in the urine of the volatile alkali combined with carbonic acid.

Occasionally, again, two of the elements of urea part company, as it were, with the other two, and enter into certain binary combinations that are also now and then detected in the urine, either alone, or accidentally conjoined with bases which they have encountered in the fluid. Thus the nitrogen and carbon uniting and separating themselves from the hydrogen and oxygen, give rise to *cyanogen* or bicarburet of nitrogen; and it is no less remarkable than indisputable that compounds of the cyanic acid, other than the cyanate of ammonia already mentioned, such as the *ferro-cyanate of potash* and even *hydrocyanic acid* in a free state, are sometimes met with in the urine. It farther happens not uncommonly, especially in certain febrile states of the system, that the nitrogen and oxygen select each other peculiarly, leaving out of the question the carbon and hydrogen, and form *nitric acid*, which, acting on the lithic acid, is the source of the compounds that have been described under the titles of the erythric and purpuric acids. Finally, if the nitrogen and hydrogen part company with the carbon and oxygen, and leave these last to unite very nearly in the proportions in which the former occurs in lithic acid, the latter in cystine, we have *oxalic acid* produced,—39.99 carbon, 53.33 oxygen,—an occasional and very formidable constituent of the urine in certain morbid states.

Under other circumstances, the kidney seems to fall short of the acidifying property it possesses as part of its distinguishing function, and then the radicals of the acids which the urine contains present themselves in an uncombined state. It is in this way, probably, that phosphorus now and then appears in solution in the urine, so that the fluid is seen to be luminous when emitted in the dark.

It is even possible that the albumen which often shows itself as a constituent of the urine, in a form of kidney disease that has lately attracted much attention, may be derived from the urea. Making urea the standard of comparison as before, if the relative proportion of nitrogen be considerably lessened, whilst the portions of hydrogen and especially of carbon are much increased, we have the elements of albumen, 15.56 nitrogen, 49.75 carbon, 8.77 hydrogen, 26.78 oxygen. In other cases the albuminous principle contained in the urine is unquestionably derived immediately from the blood, as is proved by the passage along with it of the fibrine red particles and other constituents of this vital fluid." 107.

The extract is long, but the facts which it expresses are exceedingly important, and no less important than clearly stated.

The chapter before us contains twelve sections. The first is on the—

*Discharge of Urine, which contains the Lithic Oxyde*—LITHOXIDURIA.

Our readers must be aware that Dr. Marcet described a calculus under the name of xanthic oxyde. Berzelius suspected that it was no other than uric acid, accidentally modified. But Langenbeck has lately extracted a stone which, after a very careful analysis by Liebig and Woehler, has proved to be the xanthic oxyde. Its constitution was the same as that of lithic acid *minus one atom of oxygen;* these two substances were in fact two oxydes of the same radical, the formula of lithic acid being $C^5$ $N^4$ $H^4$ $O^3$, that of the xanthic oxyde, which they of course designate with the greatest propriety lithic or uric oxyde, being $C^5$ $N^4$ $H^4$ $O^2$.

It has not been recognized either in solution or as a deposit from the urine. Dr. Willis remarks, that the lithic oxyde may be expected to occur, associated with deposites of the lithic acid. We have, in fact, but to suppose the kidney to fall somewhat short of its acidifying powers, to have the lithic oxide instead of the lithic acid (the *oxide* instead of the *acid* of urea) produced.

SECTION 2.—*Of the discharge of Urine which contains Cystine* (*cystic oxide*) *in solution or as a deposite*—CYSTINURIA.

This, which occasionally forms calculi, has been several times discovered in urine, both in a state of solution and mechanical suspension. Dr. Prout and Dr. Venables have both described cases in which they detected it. In both calculi existed. Dr. Willis has lately found it in a case in which none could be suspected. His attention was arrested by the greenish-yellow colour of the urine, its peculiar smell, and its somewhat oily appearance when passed. It reddened litmus paper very slightly, if at all; was of sp. gravity 1.030; did not coagulate by heat or nitric acid, and by standing became opalescent after thirty-six hours from the presence of a fine pulverulent matter, part of which was deposited. Acetic acid threw down a pretty copious brown precipitate, which, collected on a filter, was found to possess all the properties of cystine. This urine held a considerable quantity of the phosphatic salts in solution, which seems common; it showed no traces of lithic acid; but Dr. W. could not discover that it was deficient in urea, though, in the previously described cases, it has been so.

The best re-agents for discovering cystine in combination with alkalis are the acetic, citric, or tartaric acid; in each of which it is insoluble. The bicarbonate of ammonia immediately throws it down from its soluble combinations with potash and soda.

M. Rayer observes that the calculi formed of cystic oxyde are agglomerations of confused, semi-transparent, yellowish, and insipid crystals. The precipitate of cystine consists of hexagonal, colourless, and transparent laminæ, visible in the microscope, when its solution in potass is treated with acetic acid, or, when dissolved in ammonia, whence the crystals spontaneously separate by evaporation.

SECTION 3.—*Of the discharge of Urine, which contains the Purpuric acid, and its Salts*—PORPHURURIA.

The red colour of the sediments of febrile urine, and the pink hue of the sediment of hectic and dyspeptic urine have been ascribed by Dr. Prout to the presence of a purpurate, either of ammonia or of soda. But Berzelius concluded that the red and pink colours of urinary sediments were not caused by the intermixture of a purpurate of any base, but by the presence either of the ordinary colouring matter of the urine, or of a peculiar animal matter, which dyed the deposites in the manner of lakes. The point is not yet settled, for, as Dr. Willis observes, organic chemistry forms the low-countries of the science, the field of all the disputes that are going.

The depth of tint of the sediment would seem to depend on the quantum of the adventitious colouring principle. Sometimes the tint exists with little sediment.

"The dusky red or lateritious sediment, which recent observations have shown generally to consist of lithic acid combined with an animal colouring matter, is the well-known herald of the abatement of febrile and inflammatory action in the system, and is always looked for anxiously in cases of danger by the attentive pathologist. The depth of tint of the sediment let fall under such circumstances, has even been observed to afford a kind of criterion of the intensity of the symptoms, the deep dusky red sediment showing itself during and especially on the abatement of high inflammatory fever in vigorous subjects; the paler coloured and fine pink precipitates being associated with action of a less energetic kind, with the low fever of local organic disease, or hectic, as it is called.

A purpuric state of the urine (without regard to the nature of the colouring principle) in a less marked degree appears to be almost habitual to certain individuals of excitable and delicate constitution, among whom slight errors of diet, exposure to cold, and even an ungenial state of the atmosphere, though no chill has been suffered, are observed to produce it. Probably the purest specimens of the bright pink sediment that are ever met with, are deposited from the urine of some dropsical subjects, and of those who are labouring under chronic visceral affections especially of the liver. There seems no reason, however, to conclude that visceral diseases are present as the causes of these pink sediments in every instance in which they occur; on the contrary, they are frequently observed accidentally in cases where there is no room even to suspect the existence of organic disease, and in which none certainly exists.

Occurring occasionally and in conjunction with obvious causes of general excitement, or of particular local derangement, a purpuric state of the urinary secretion is to be regarded as of just as much but not of more importance than accidental lithic states of this product. When it presents itself in a high degree, however, and habitually for any length of time, there are just grounds of alarm; a deranged condition of the general functions, depending in all probability on some latent organic mischief of a local nature, that may ultimately bring the patient's life into jeopardy, being indicated." 114.

There need not be said much on the subject of treatment. When an attendant on febrile excitement the remedies for the latter are usually the remedies for it. When the general features are those of the lithic diathesis, the latter becomes the subject of treatment. In short, the object is to determine the cause, organic or functional, of the affection, and to act accordingly. Pink coloured lithic deposites certainly should not be neglected. They have formed, in two instances, the nucleus of a stone.

SECTION 4.—*Of the discharge of Urine which contains a Salt of the Oxalic acid,*—OXALURIA.

Dr. Willis commences this Section by observing:—

"Lithic acid under the prolonged action of nitric acid and of chlorine, is partly converted into oxalic acid. Liebig and Woehler found that when lithic acid was boiled with the superoxide of lead it was resolved into urea, alantoin, carbonic acid, and oxalic acid. I have besides shown, in the general remarks introductory to the different sections of the present chapter, that the proportion of carbon which exists in lithic acid, united to the proportion of oxygen which occurs in cystine, form as nearly as may be the combining quantities of the elements of oxalic acid. I do not, therefore, find any peculiar difficulty in accounting for the occasional existence of oxalic acid as a product of the renal secretion. It does not seem necessary to suppose that this substance should be introduced among the ingesta: its elements exist in urine, and the chemic art of the kidney may, and undoubtedly does, suffice at times to combine them into the compound in question. Nevertheless it remains true that certain articles of food, which are pretty generally used in some countries, contain oxalic acid; and the experiments of Woehler have put it beyond doubt that it is one of the few acids that make their way into the torrent of the circulation, and are then eliminated both free and combined with a base from the system by the kidney. The urine contained in the bladder of a dog, killed eight hours after having had two drams of oxalic acid mixed with a quantity of meat and bread given to it, was found to deposite a precipitate on cooling, which bore an exact resemblance to that formed by the triple phosphate. The clear urine mixed with a little of a solution of nitrate of lime gave a farther deposite, having the same external characters. On examination both of the deposites were found to consist of oxalate of lime. The articles of food used by man which contain oxalic acid in largest quantity are the sorrel, (Rumex acetosa), so much eaten by all classes in France, and consumed to some extent by the upper ranks in England as an agreeable vegetable; the tomata, (Solanum lycopersicum), of which many individuals are passionately fond; and the leaf-stalk of the rhubarb plant, (Rheum palmatum,) which in the spring and early summer months is consumed in large quantities made into pies and puddings by the community at large in England." 118.

M. Rayer appears to doubt the influence of sorrel in producing oxalate deposits. Yet evidence seems in favour of the supposition. The oxalate of lime has not often been observed as a precipitate from the urine. But Mr. Henry Brett has lately published an interesting case of it. Dr. Willis thinks that when the urine is more carefully and more scientifically examined, deposites of the oxalate will be found not to be rare. It probably, however, occurs most frequently as an ingredient in amorphous urinary sediments. It has been detected in company with the amorphous lithic acid sediment—and has been confounded with the lithate of lime.

The oxalate diathesis is probably intimately connected with the lithic. On this hint the treatment is founded, for the same general plan must be pursued as is required for the latter. Those who labour under it should, of course, give up those vegetables which contain oxalic acid.

5. The next Section is appropriated to the discharge of urine, which contains albumen (as a derivative from urea?) We shall defer what our author says of albuminous urine, until we take that up.

Section 6.—*Of the discharge of Urine which contains the Elements of Urea in the shape of Carbonate of Ammonia.*

The elements of one atom of urea, combining with those of one atom of water, constitute one atom of carbonate of ammonia, a combination which implies decomposition both of urea and water, and occurs very readily in high temperatures.

This change, the common consequence of decomposition without, may occur in the kidney itself, from which it is directly eliminated by an altered mode of secretion. Dr. Graves was the first who demonstrated this fact. He has related two instances. The first was that of a patient labouring under bad continued fever with petechiæ. The urine abstracted from the bladder, which showed no signs of disease, within two hours after it had been completely emptied, was found strongly ammoniacal. The second case was that of a powerful labourer, who fell dangerously ill of fever and anasarca, from having worked up to his knees in water during cold weather. The urine here was of a pale straw colour, and deposited the phosphatic salts on standing; it smelt strongly of ammonia, and effervesced briskly on the addition of an acid, when a sample of it, obtained from the bladder within so short a time as half an hour after this viscus had been completely emptied, was examined. This patient died. The bladder was found perfectly healthy. The kidneys were enlarged, and turgid with blood. The liver was much diseased. The urine was without a trace of urea. In a case of ascites, quoted by Nysten, the urine contained much carbonate of ammonia, but no urea; and, in two cases which were under treatment for the honey diabetes in the Royal Infirmary of Glasgow, by means of animal food and opium, the urine which had been highly ureous, became suddenly alkaline, and was found to contain a large quantity of ammonia, but no urea. In these cases he combines the elements of urea so as to constitute the carbonate of ammonia.

Dr. Willis goes on to remark, that in certain irritable states of the system, when the urine is secreted copiously, and with a disposition to throw down the phosphatic salts, circumstances in which the fluid is often neutral, or acid only in the very slightest degree, any additional irritation, such as that produced by the introduction of a bougie or catheter into the urethra, will often suffice to turn it positively alkaline.

The state of the urine in typhoid fever, with regard especially to its acid or alkaline reaction, is often advantageously consulted as an index of the progress of the disease. In the earlier stages the urine is acid, and as the disease advances it becomes neutral, and then alkaline; as the disease declines, on the contrary, the urine from alkaline becomes neutral and then acid. The return to the acid state is always a favourable sign, and may sometimes enable us to give a flattering prognosis when there is nothing else in the state of the patient that betokens improvement.

We need hardly observe that ammoniacal urine can only be remedied through the system at large. To treat it is to treat the constitutional state of which it is a consequence and index. But it is important to be aware, that the urine may be secreted ammoniacal, for when this condition is observed it is usually attributed to putrefactive fermentation of the fluid in the bladder.

In certain chronic diseases of that organ we have indeed ammoniacal urine of the most offensive description evacuated. There, the ammonia seems really due to rapid decomposition of the urine effected by the putrefactive ferment of a diseased vesical mucus. In many of these cases if the bladder be carefully washed out as a preliminary, urine may be withdrawn by a clean catheter, that will show acid reaction. But this latter circumstance is decisive of the fact that the urine in this case was not secreted ammoniacal, a characteristic and diagnostic feature. Healthy acid urine, continues Dr. Willis, uninoculated by such a ferment, is a fluid that is by no means greatly disposed to run into putrefaction; we often see it withdrawn in cases of retention, after having been pent up in the bladder for two or three days, perfectly unchanged; and I have kept the highly acid urine of a patient labouring under calculus of the kidney in an open vessel for more than a fortnight, in a temperature varying between 50° and 65° F. without its undergoing any change. The ammoniacal urine which is the product of a new combination among the elements of urea at the moment of its formation, is not particularly and otherwise offensive—it has the smell of ammonia superadded to its own urinous odour. That which results from putrefactive decomposision is fetid and disgusting in the last degree.

Section 7.—*Of the discharge of Urine containing the Hydrocyanic and Ferrocyanic Acids.*

"It has happened in more than one instance in which a salt of iron had either been taken in the way of medicine or accidentally, that the urine voided has presented a blue colour, from containing the compound of ferrocyanic acid and iron which is commonly called prussian blue. Thus Dr. William Batt of Genoa, observed in a girl who had been taking six grains of the æthiops martialis or black oxide of iron daily for a few weeks, on account of some stomach complaints, that the urine evacuated was of a blue colour. The fluid collected and set aside deposited upon the bottom and sides of the recipient a quantity of sediment of a beautiful blue colour. This sediment analysed by Professor Mojon was found to consist of the prussiate of iron. M. Julia Fontanelle has given the histories of two cases in which urine was discharged having a blue colour. The first occurred in the person of an old man aged 82, who, on the second day of an illness caused by an acute affection of the urinary passages, began to pass urine which was thick and ropy, and of a deep blue colour. The ropiness was owing to the presence of a plentiful admixture of albumen or gelatine, the colour to that of the hydrocyanate of iron combined probably with soda. This patient had taken no preparation of iron. But as oxide of iron has been shewn to be a constant constituent of urinary sediments deposited during febrile states of the system, its presence in the colouring compound is readily accounted for. The second case happened in a lad of 15 who was suffering from violent colic, in consequence of having swallowed a quantity of ink by mistake. The urine when first voided was of a greenish blue colour, which after the lapse of an hour deepened into a rich blue. The tint became still more intense (probably from an additional quantity of precipitate being thrown down) when a few drops of the persulphate of iron were added to the urine. The sediment of the urine was found to have all the properties of prussian blue." 130.

Dr. Willis quotes some other cases to which we need not advert. The urine is generally stated in these cases to have been thick and ropy, and to have contained albumen or gelatine. In one case it contained sugar. In another it shewed traces of urea.

The presence of cyanic acid was only discovered in these cases from its having accidentally met with iron and given rise to a blue colour.

"There is every reason to believe that the cyanic or hydrocyanic acid may often exist in urine, and pass unsuspected. One instance indeed and only one so far as I am aware is recorded, in which prussic acid, free and uncombined, as

I read the various reports of the case, has been detected in the urine. This instance occurred to the distinguished Italian chemist Brugnatelli.* The patient who was the subject of the observation, was affected with dropsy, (an *ascites,* I believe, from disease of the liver,) and the urine contained almost no urea. In M. Cantin's case, free hydrocyanic acid was also ascertained to be present, along with the compound of this acid and iron.

There can be little doubt but that the urine, were it properly examined, would be found to contain prussic acid in a variety of morbid conditions of the general system. Hydrocyanic acid is a product of the destructive distillation of animal matters generally, of blood and lithic acid in particular; and we know enough of the chemic powers of the kidney to warrant our admitting its ability upon occasion to separate the elements of the hydrated cyanate of ammonia, or urea, combined as cyanic acid and water, just as they are occasionally eliminated in the shape of bicarbonate of ammonia.

I am not aware that the presence of hydrocyanic acid or of the prussiate of iron in the urine has been attended with any peculiar consequences. Dr. Batt's patient did well. So did both the cases seen by M. Julia. The urine in the cases generally, however, seems to have presented other evidence of being in a morbid state, and in some of them at least, was undoubtedly connected with constitutional derangement, probably with organic lesion, that would have received the most careful consideration from an enlightened pathologist. The albuminous, or as they are sometimes improperly styled, gelatinous states of the urine, point to the existence, in some cases, of inflammatory action in the system, a circumstance which is also farther proclaimed by the plentiful traces of iron encounteied, a substance only met with in quantity in the lateritious sediments of inflammatory and febrile urine. These considerations taken in conjunction with every other circumstance would influence, and probably to a certain extent guide, our mode of treating such cases." 133.

SECTION 8.—*Of the discharge of Urine containing Carbonate of Lime in Solution or as a Deposite.*

The carbonate of lime, though it forms an occasional constituent of urinary calculi, has not, to Dr. Willis's knowledge, been observed, either as a solution or a deposit in human urine. Yet he does not doubt that it occasionally exists, though held in solution by an excess of carbonic acid, which also aids in keeping the phosphatic salts dissolved, or disguised from intimate admixture with these and other precipitates, it must always be detected with difficulty.

SECTION 9.—*Of the discharge of Urine which is luminous or phosphorescent.* —PHOSPHORURIA.

The acidifying influence of the kidney on the elements or bases presented to it, may sometimes be imperfectly exerted. The phosphorus in the urine may be in this predicament. Dr. Willis states that there are actually several instances on record, in which the urine has been voided directly from the bladder, either generally luminous, or having luminous points intermingled with it. Thus: Dr. Jurine, then in his usual health, whilst making water one dark night in a corner, observed that his urine bore numerous shining

* "I have searched in vain for a reference to the original record of the case by Biugnatelli. Almost the only place I have not looked into in the hopes of finding its history, is the Journal edited by him, I think at Pisa, in 4 vols. 8vo. 1791-2."

points along with it in its current. The boards down which it flowed, and the leaves upon which it fell, also glimmered with many luminous particles or spots the size of a small lentil. The light continued for about thirty seconds and then disappeared. To make sure that the luminous points he observed were voided with his urine, the Doctor passed a little of it into the palm of his hand, and in this he observed several of them floating about. He closed his hand and hurried home to examine the small quantity of fluid he could carry in this way; but even with the assistance of a magnifier he could discover nothing. Doctor Jurine was living in his usual temperate way when this happened, and though he was on the watch for a recurrence of such a phenomenon, he did not perceive his urine to become phosphorescent for a long time. At the interval of a year, however, the same thing happened again.

Dr. Willis cites, in addition to the preceding, the cases of Dr. Guyton, of Autun—of M. Pictet, of Geneva—of Dr. S. Reisel—and of Dr. L. Pettenkover, all of whom made water very luminously. These enlightened individuals were all men of science, yet Dr. Pettenkover diffidently owns to a tender sentiment towards beer. Dr. Willis asks whether, in those jolly people, who now and then expire of spontaneous combustion, the phosphorus of the body is properly eliminated by the kidney ?

Section 10. *Of the discharge of Urine of various abnormal and remarkable colours.*

The nature of the ordinary colouring matter of the urine is still imperfectly known. Nor is it determined whether the abnormal tints of the urine result from modifications of the colouring matter in question.

"When the colour of abnormally tinted urine depends on the presence of an alkaline purpurate, for a cyanate or phosphate of iron, we conceive the colouring matters developed at the expense, or in consequence of the decomposition, of one or other of the ordinary ingredients of the urine. But instances of red, blue, green, and black urine have occasionally been observed, the peculiar colour of which could not be referred to the existence of any purpurate, or of any combination of iron with the cyanic or phosphoric acid. Of this description appear to have been the varieties of urine observed by many of the ancient physicians, and particularly described by Actuarius, who flourished towards the end of the thirteenth century, in his work 'De Urinis,' under the titles of *Urinæ venetæ, lividæ et nigræ.* The kind of urine characterized as *Urina veneta,* appears to have been of a slate-gray colour, for the tint designated by the epithet he informs us may be imitated by mixing equal parts of ink and white lead together. The word *venetus* was afterwards used however to signify a deep blue." 139.

The recent researches, continues Dr. Willis, of Marcet, Prout, and Braconnot have, however, placed it beyond doubt that some deep brown or black, and blue coloured urines owe their peculiar tint to the presence of a new proximate principle, which with reference to a dark brown or black urine was spoken of under the title of *Melanic acid* by Dr. Prout, and by Braconnot under that of *Melanourine.* In giving the chemical history of a blue urine, the last-mentioned distinguished chemist speaks of the colouring matter under the name of *Cyanourine.*

Cases of black urine are rare. Dr. Marcet has left an account of two. The first was that of a young female subject to febrile and hysterical attacks, during which the urine assumed a deep purplish brown or black colour. The second case was that of a child seventeen months old, whose urine from the time of his birth had been observed to stain his napkins of a dark purple colour. At nine months of age, the urine was occasionally observed when first discharged to be clear and almost colourless, but gradually to acquire a dark colour like that of port wine, which deepened continually by standing until it became black. At the age of seventeen months, the child was robust and lively. The urine was carefully examined. In one specimen, the deep colour seemed to be due to the action of ammonia, spontaneously evolved, upon a peculiar principle or matter held in solution by the urine. Dr. Prout regarded this as of the nature of an acid, and accordingly named it *melanic acid.* He found it to bear a closer analogy to lithic acid, or to some of its products engendered by the action of nitric acid, than to any other principle contained in the urine. This specimen of urine remained without further change for a period of seven years, not even depositing any sediment in all that time. A second specimen became alkaline and putrid. A third, colourless at first, became ammoniacal, and acquired a pale claret colour.

" In his account of the chemical examination of some specimens of blue and black urine, M. Braconnot speaks of the colouring principles under the titles of Cyanourine and Melanourine. But, instead of regarding them as of the nature of acids, with Dr. Prout, he found these new proximate principles to have the property of bases, to combine with acids as the weaker alkalis do, and to form compounds, which as subsalts were of a brown, and as supersalts of a brilliant carmine colour. Berzelius remarks, that the melanic acid of Prout is very analogous to the black pulverulent substance, insoluble in alcohol, which is developed when the extractiform constituents of urine are submitted to the action of concentrated acids. The substances named melanic acid and melanourine seem also to bear a strong resemblance to those produced by the action of the hydrochloric acid on fibrine and albumen. If pure fibrine be acted on in this way, the colour of the solution is a rich blue; if it has not been quite pure the tint is of a greenish black, or black." 145.

Dr. Willis refers to two other cases of black urine. In the first, the sweat was black too. In the second, the discoloration of the urine disappeared during the exhibition of the decoction of uva ursi and carbonate of soda.

SECTION 11. *Of the discharge of Urine of various peculiar and abnormal odours.*

Several substances, medicinal and dietetic, are well known to impart an odour to the urine—turpentine that of violets—asparagus a disgusting one. Dr. Willis has observed, that the urine of those who have weak digestive powers very constantly partakes in a greater or less degree of the odour of the higher flavoured articles of food they consume. The odour of boiled beef, of stewed celery, of ale and beer, &c. &c., may always be detected in the urine of such individuals.

The smell of the urine, he continues, is also affected to various extents in the course of different diseases. It has sometimes been remarked of a musky odour; occasionally it has the peculiar smell of mice, which seems to pervade the whole body in certain cases of typhoid fever. In other cases it is simply fetid and sickly. He has in particular very frequently observed the urine to have a most offensive odour in children who were labouring under affections of the digestive apparatus.

Dr. Willis promises to pursue the investigation of this part of the subject, and anticipates something from it.

Section 12.—*Of the discharge of Urinewhich contains Silica in solution or as a deposit.*

Silica, a sparing ingredient in urine, has been detected in a few urinary calculi. Given to animals, in combination with potash, it finds its way out of the system by the kidney.

"Masses of siliceous matter, said to have been passed from the urinary bladder, are very frequently sent or brought to medical men and chemists for analysis. There are few collections of calculi in which a box of *siliceous gravel* will not be found. In that of the Royal College of Surgeons in London, for example, I observe one. Dr. Venables believed that he had seen siliceous matter voided by the urine in two instances. In the one, the concretion was of some size, and bore considerable resemblance to a tooth, to which it was likened by Dr. Prout when it was shown to him. In the other, the siliceous matter was like river or sea-sand. Medical practitioners have repeatedly had considerable masses of various mineral substances presented to them as urinary calculi, which they have often been able to pronounce at a glance to be ordinary rolled pebbles of the surrounding country. A friend informed me lately, that within a few weeks he had had, I think, three samples of siliceous *urinary gravel* transmitted to him for examination, which in each case consisted of pieces of quartz; and I know that both Dr. Bostock and Dr. Christison have oftener than once been requested to ascertain the chemical composition of certain masses of quartz and flint which were said to have been voided from the bladder.

There is every reason to believe that Dr. Venables was imposed upon by his patient, as, in the other instances quoted, imposition was attempted. It has often been remarked, that the so-called *siliceous gravel* is always of a description in relation within the geological structure of the district in which it is said to have been discharged. Some of the specimens have even had portions of other minerals, with which quartz is known to occur associated, adhering to them. This was the case with at least one of the specimens sent for Dr. Bostock's examination. Patients, and especially female patients, often show a singular taste for having diseases unlike all the rest of the world; and most of the instances in which these siliceous concretions were said to have been voided, occurred in females." 147.

We now arrive at a new chapter and a highly important subject—that of albuminous urine. We have hitherto almost confined ourselves to Dr. Willis. We shall now turn to Rayer and to Christison, particularly to the latter. The chapter of Dr. Willis' before us is intituled :—

MORBID STATES IN WHICH CERTAIN MATTERS BEING CONSTITUENTS OF THE BLOOD ARE CONTAINED IN THE URINE.

Dr. Willis starts with the remarks :—

"When we observe the rapidity with which fluids in general, and many saline, odorous, and colouring matters taken into the stomach are rendered by the kidneys, when we consider the great vascularity of these organs, and the freedom of communication that must exist between the arterial exhalents and the uriniferous tubules, we are almost prepared to expect that the blood, escaping from its proper channels, will occasionally make its appearance in the urine. And accordingly we find that under a variety of circumstances, some of which can scarcely be regarded as of a proper pathological nature, one or other of the elements of the blood,—the albumen, the oil, the fibrine and the red globules show themselves either severally or associated, mingled with the product of the renal function.

Chemists are now generally agreed in considering the proximate organic principles named *albumen, fibrine,* and *caseine,* as mere modifications of one and the same element, which is conveniently and with great propriety indicated under the common epithet of albuminous. The urine, if the reports of highly respectable authorities are to be taken, occurs impregnated with the albuminous principle in each of the states constituting the three modifications that have been mentioned. But the existence of caseine as a constituent of the urine under any circumstances, has lately been called in question by M. Rayer, who has shown that the evidence upon which its presence was admitted is defective." 150.

He divides the chapter into three sections, in the first of which he examines urine which has the albumen of the blood mixed with it—in the second, he treats of urine having the oily and albumino-fibrinous elements of the blood mingled with it—and in the third, he considers urine which has all the elements of the blood mingled with it.

Dr. Christison will soon be found to connect albuminous urine very closely with granular degeneration of the kidneys. His work is made up of six sections, followed by a series of illustrative cases.

His first section is devoted to the pathology and morbid appearances of granular degeneration—his second to its symptoms and history—his third, to the secondary diseases connected with granular degeneration of the kidneys—the fourth, to the causes of granular degeneration—the fifth, to prognosis—the sixth, to treatment.

We shall first present an account of the more general remarks of Dr. Willis, and of M. Rayer, then pass to the more special ones of Dr. Christison.

Section 1.—*Of the Discharge of Urine, having the Albumen of the Blood mingled with it*—Sero-Albuminous Urine.

Very slight causes, observes Dr. Willis, especially such as interfere with the due performance of the process of digestion, have frequently the effect of rendering the urine even of a person in perfect health slightly turbid. In higher degrees of disturbance of the digestive function, and indeed under many circumstances not adequately understood, the urine is secreted opalescent and possessed of the appearance of whey.

" The milkiness in such cases has been ascribed to the presence of albumen. Dr. Bostock even came to the conclusion that albumen may at times be detected by appropriate tests in the urine of the great majority of individuals, apparently in perfect health. In his own person he had observed the quantity to be considerably increased by very trifling causes. I have in very many instances observed the urine secreted under the circumstances indicated, to be opalescent or milky; indeed, there are probably few men alive, who when they have been compelled to exert themselves immediately after a full or hurried meal, have not noticed their urine to become turbid, and as if mixed with a little chalk or milk. I have not, however, found that the cause of the milkiness or want of transparency in these cases, was due to the presence of albumen. There may, it is true, have been rather more than the average quantity of vesical mucus diffused through the urine, but the microscope showed that the opacity depended upon the suspension of an amorphous precipitate, which the proper reagents afterwards proved to consist of the ordinary earthy salts of the urine. Albumen, indeed, is by itself incompetent to cause milkiness such as is observed in these cases; a solution of simple albumen in water, or water impregnated with the saline constituents of the urine, is transparent. By the application of heat or the addition of a drop of nitric acid, such a solution immediately becomes turbid. The turbid urine in question, exposed to heat becomes opaque in an increased degree; but the addition of a drop of nitric acid, either before or after it is boiled, gives it complete transparency. This subject has been already touched upon in the section on Earthy Urine (Ceramuria). The proper inference from this diversity of experience is, that albumen is not always the cause of the milky or turbid urine rendered under the circumstances specified, by individuals otherwise in good health." 152.

M. Rayer directs attention, with some degree of earnestness, to the same fact—a fact which is, perhaps, occasionally lost sight of by hasty practitioners. M. Rayer's remarks, indeed, deserve a little further consideration.

If, he says, albuminous urine which is discharged *alkaline*, or soon afterwards becomes so, is exposed to the action of heat, it is not in general coagulated, sometimes indeed, if previously transparent, it exhibits no turbidity. If the quantity of albumen is considerable, it assumes a milky tint. If a few drops of nitric acid are added, the albumen is immediately coagulated and precipitated, while the superabundant urine is transparent and of a reddish colour, from the action of the acid on the colouring matter or on the uric acid. If the addition of nitric acid be extremely small the albumen will probably not be coagulated, but if the quantity of acid is increased the desired effect ensues.

From several experiments made by M. Guibourt and M. Rayer for the purpose of determining the cause of this phenomenon, the latter concludes that, nitric acid in minute quantities, acetic acid, and phosphoric acid prevent coagulation of albumen by ebullition, but not precipitation by a further quantity of nitric acid. As urine might contain both albumen and a little free phosphoric acid, it follows that nitric acid is a better test than ebullition. It is better, however, to combine the two, and to examine the coagulated or precipitated matters in the microscope besides.

M. Rayer, as we have already stated, insists on the fact that alkaline urine may become turbid on the application of heat in consequence of the precipitation of the phosphates; the addition of nitric acid renders the urine clear. If nitric acid be added to it prior to the ebullition, turbidity is equally prevented. M. Rayer has often seen men of high general attainments commit the mistake of supposing that such urine was albuminous, in consequence of the effect of heat upon it.

We return to Dr. Willis. He goes on to remark that the most simple and common form of albuminous urine is that which is met with in dropsical affections, especially in anasarca.

In this class of cases, he continues, the albuminous principle is mingled with the urine in the state in which it occurs in the serum of the blood and the white of the bird's egg; and would escape observation but for the power possessed by heat and certain chemical reagents of causing its coagulation and precipitation in a solid form. In its sensible properties albuminous urine varies greatly, and does not always differ essentially from the healthy fluid. Sometimes, perhaps most generally, it is paler in colour, secreted in larger quantity, and froths more when passed in a full stream into the utensil than usual. In such cases the specific gravity of the urine is uniformly low. In other cases, however, in which the urine is albuminous, the quantity elaborated is below rather than above the proper standard, and the fluid is high coloured, and of great density. This happens in the course of different acute inflammatory diseases. It is worthy of remark, that in some of the forms of disease in which the urine finally becomes copious and of inferior density, it is in the first instance scanty, and of higher specific gravity than wont. The anasarca that succeeds scarlet fever is very commonly accompanied in its invasion by dense, high coloured, and scanty urine. Brought to the temperature of 120°, albuminous urine begins to grow turbid, and as the temperature rises, to deposit coagulated albumen in clouds or flakes, which subside to the bottom. The amount of abnormal impregnation detected in this manner varies extremely; in some cases it is so trifling as to be barely perceptible, in others it is so great, that nearly the whole portion of urine submitted to experiment runs together into one solid mass, as the serum of the blood would do. Nitric acid dropped into this urine also causes a precipitate, which is abundant in proportion to the quantity of albumen it contains.

In the pale-coloured albuminous urine of low specific gravity there is always a deficiency of urea; and it is interesting and important to know that this deficiency is uniformly in proportion to the abundance of the albumen, a fact which plainly points to a connexion between these two proximate organic principles, and shows the morbid state proclaimed by the disappearance of the one and the appearance of the other, to be of the nature of that which he has described under the head of ureo-albuminous urine. But, in the deep-coloured albuminous urine of average or high specific gravity, the urea is not necessarily deficient in quantity. Dr. Willis has detected it by actual experiment, at the least, in the proportion in which it exists in health, in a variety of cases—in an instance of suppuration of the kidney, probably induced by the presence of a calculus in its pelvis; in a case in which the disease had been hæmaturia, but the urine at the time the examination was made, although highly albuminous, contained no red globules in anasarca after scarlatina, &c.

M. Rayer directs attention to the necessity of examining both the albuminous precipitate *and* the urine from which it has been separated, in order to appreciate the existence and the amount of urea. That principle exists in both, although some have overlooked it in one, and many in the other.

The turbid portion of albuminous urine, if examined in the microscope, exhibits lamellæ of membranous appearance, of variable dimensions, and of irregular form. Occasionally whitish, they are usually of a more yellow tint. Their surface is areolar; they are semi-transparent, yet not always so, for some may be perfectly opaque. M. Rayer has observed this lamellar appearance in those cases more especially in which the albuminous urine was acid, and deposited crystals of uric acid.

Dr. Willis glances at the pathology of the simple albuminous urine, especially of that which occurs with general dropsy. We present his brief summary of opinions. They will be an useful hereld to the more exclusive sentiments of Dr. Christison, to which we shall soon pass.

"Whilst some," he says "maintain that the appearance of albumen in the urine of dropsical patients is the never-failing indication of organic mischief going on in the kidney, others have insisted that this circumstance was often indicative of simple febrile excitement of the circulating system, of some general or local inflammation, of functional derangement of the kidney at most, and sometimes not even of that, the albuminous impregnation of the urine being the mere effect of a general effusion of a serous fluid into all the cavities and interstices of the body. Mr. Cruickshank in particular held albuminous urine to accompany 'every case of increased action of vessels, more particularly when this was of the inflammatory kind,' and believed that anasarca (which he calls *general dropsy*, and which he obviously considers as a disease dependent on increased vascular action) may be distinguished from the dropsy produced by a morbid condition of one or other of the viscera, from the circumstance of its always being accompanied by an albuminous state of the urine. Dr. Wells concludes his truly excellent paper, 'On the dropsies that follow scarlet fever,' by advocating the practice of blood-letting in such cases, regarding them as connected with a general inflammatory state of the system. Dr. Blackall, too, whose experience and candour deserve and secure our highest consideration, holds the presence of albumen in the urine of dropsical patients to be a certain indication of the existence either of a general inflammatory diathesis, or of some distinct local inflammation which requires antiphlogistic measures—the abstraction of blood, low diet, &c., for its subdual.

The same idea of increased vascular action as the cause of albuminous urine, has been carried still farther in very recent days, but with a progressive disposition to localize the inflammation, and to attach it to the kidney. This is the general tendency of that section of Dr. Bright's recent admirable work, which is devoted to the consideration of 'Dropsy with an albuminous state of the urine.' The cases and observations of the Drs. Gregory and Christison have a similar bearing. In six of the fifteen beautiful plates of his magnificent work now in course of publication, Dr. Rayer has given representations of the kidneys in various *nephrites albumineuses*, or inflammations of the kidney with secretion of albuminous urine.

It is difficult to resist such high authority; nevertheless, several distinguished practitioners have opposed themselves to the fundamental notion espoused by all the names that have just been mentioned. Dr. Graves, for instance, positively denies that the albuminous state of the urine in dropsies always or even generally depends on structural change in the kidneys. He had seen so many cases in which the albuminous state of the urine entirely and speedily disappeared under the influence of proper treatment, that the only inference he could draw was, that this state frequently depended on mere functional derangement of the secreting organ. Dr. Graves was even led to prescribe opium and animal diet in some cases of dropsical effusion with albuminous urine, with the very best effects. Dr. Mateer of Belfast was led to believe that the albuminous urine of certain dropsies was sometimes the effect of stimulating tonic and saline medicines. Mercury is well known occasionally to have the effect of causing the urine to be secreted loaded with albumen. The late Dr. M'Intosh of Edinburgh and Dr. Elliotson have also expressed themselves against the notion of structural disease as the general cause of serous urine in dropsy of the cellular tissue. Legitimate deduction from my own experience in numerous cases of anasarca succeeding scarlet fever and exposure to cold, which I have had an opportunity of treating at the Royal Infirmary for Children, would also lead me to conclude that structural disease of the kidney could not have been the cause of the albuminous state of the urine which I found almost invariably to accompany the progress of the disease. Finally, it is well known, that the urine is albuminous in the course, and especially as M. Martin-Solon has shown, on the crises, of a great number of acute diseases. The following Table from the work of the writer just quoted, exhibits the results of his experiments in this direction:—

| DISEASES. | NUMBER OF CASES. | URINE COAGU. | URINE NOT COAG. | RESULT. |
|---|---|---|---|---|
| Intermittent Fever.. | 8 | 7 | 1 | All cured |
| Typhoid Fever...... | 23 | 19 | 4 | 21 do. 2 dead |
| Rubeola .......... | 7 | 5 | 2 | All cured |
| Variola .......... | 11 | 2 | 6 | Ditto |
| Scarlatina ........ | 23 | 22 | 1 | Ditto |
| Pleuro-pneumonia .. | 24 | 5 | 2 | 19 do. 5 dead |

In the generality of rheumatic cases, also, the urine was found coagulable by heat or nitric acid; but no exact account of these having been kept, it was impossible to insert them in the Table.

Far from being the herald of organic disease of the kidney, and therefore of despair in every case, albuminous urine would consequently appear to promise a happy termination to many formidable diseases, and to be a sign that may be often looked for with an expectation of proving advantageous." 158.

Dr. Willis grants that sero-albuminous urine is a very unfavourable item in those cases of disease of the kidney, in which the symptoms are referred to the bladder and urethra. Of this there cannot be a doubt. Yet we have seen cases of this description, of the gravest character, proceed for a length of time without terminating fatally, nay even exhibit ultimate amelioration. It must be owned that such fortunate instances are rare.

Dr. Willis concludes that sero-albuminous urine of the kind we are discussing, however, is under circumstances a symptom of very unfavourable import. In those interesting and intractable cases of kidney disease in which the symptoms are referred to the bladder and urethra, the urine is constantly sero-albuminous. I had very lately a gentleman under my care whose urine was extremely acid, of high rather than low specific gravity, mixed with a large quantity of pus and loaded with albumen. The whole of the symptoms are so nearly like those of stone, that I still flattered myself, against my better knowledge, that the bladder might contain a calculus, until, having allayed the irritability of the urethra by appropriate treatment, I was enabled to introduce a sound and ascertain that this sac was empty, and for aught I could discover to the contrary, quite healthy.

From all that goes before, it is obvious that sero-albuminous urine is indicative of no particular malady; on the contrary, it occurs as a feature in a great variety of diseases, and must be met by the treatment appropriate to each of these. Generally speaking, the antiphlogistic plan,—bleeding from the system, from the region of the kidney, or from the neighbourhood of any organ that appears to be particularly affected, may be considered as the rule. To this, however, there are many exceptions.

We think that we must now desert Dr. Willis and scan more closely the pages of Dr. Christison.

As he divides the granular degeneration of the kidney into three stages, and attempts to appropriate its proper alteration of the urine to each, it would be useless endeavouring to amalgamate his observations with those of the gentlemen with whom we have associated him.

Dr. Christison commences by observing:—

"The disease has been variously divided by authors into several forms, by *Dr. Bright* into three, by *M. Martin Solon* into five, by *M. Rayer* into no fewer than six. Of these varieties some, or even all, have been considered mere stages of the same morbid affection. The latter view is exceedingly doubtful. For not merely does the disease thus supposed to be one and the same in nature present a very great diversity of anatomical characters,—the kidney being sometimes much enlarged, sometimes excessively shrivelled, at one time little firmer than recent brain, at another harder than the hardest tubercular liver, in some cases composed of a smooth, homogeneous mass, in others finely granulated like herring-roe, and in others coarsely tuberculated, so as to present somewhat the appearance of being thickly studded with peas;—but likewise the several opposite characters here enumerated may occur in different cases, where the amount of suppression of the solids of the urine during life, and the extent of disorganization of the healthy structure of the kidnies, which are the best measures of the stage or progress of the disorder, seem entirely the same. It is highly probable then, that the various forms mentioned by authors do not exactly belong to the same morbid formation.

When pathologists shall succeed in arranging these forms according to their exact relationships and true differences, some obscure points in the history of the disease, more especially in its symptomatology, will in all likelihood be cleared up. Meanwhile however such an arrangement is impracticable. And the resemblances are such between the manifestations of all the forms during life,—the leading characters of the urine in particular are so much alike, and above all there is such an identity in the secondary diseases to which they lead, and which are the direct sources of danger to life, as well as the chief subjects of treatment,—that there can be no impropriety in considering the whole forms for the present under one head. And to avoid pathological error as far as possible, it may be preferable, in following out the disease in its progress through successive stages, to look rather to the destruction of the healthy structure, than to the morbid deposit." 4.

If, upon the one hand, we see, as we certainly do, albuminous urine without appreciable organic alterations of the kidney—and, if, on the other, we find it accompanied with lesions which differ too widely in appearance to permit us to suppose that they have resulted from the same kind of morbid action, it does seem consistent with analogy and reason to believe that several diseases, nay that some forms of functional disturbance of the kidney may have a common symptom. This is the obvious deduction—the one that squares the best with the facts. If the progress of knowledge reduces these various modes of action and varieties of lesion to a simple formula, well and good. But it is not philosophical nor is it safe to anticipate. We must frame our opinions on what we know, not on what we suspect; with the progress of facts will come the progress of opinions also. When Dr. Bright, for instance, found one out of every six patients taken indiscriminately presented traces of albuminous urine, and concluded that organic alterations of the kidney obtained in that proportion, he drew an inference which, however agreeable to Dr. Christison's views, is by no means admitted by the great majority of practical and scientific physicians.

The idea of looking to the destruction of the healthy structure rather than to the nature of the morbid which encroaches on it, is convenient, perhaps, but loose. Apply the same mode of investigation to the lung. All its maladies would be attended with a common symptom of dyspnœa—with a common effect, impaired aeration of the blood. If, reasoning back from these, we concluded on a common lesion, or argued that dyspnœa and imperfect aeration of the blood constituted a disease per se, we might find ourselves seriously embarrassed in the attempt to reconcile facts with our hypothesis. This very difficulty has occurred to Dr. Bright in the case of the kidney. Is it conceivable that one out of six patients taken indiscriminately should have granular degeneration, or indeed any lesion of the organ?

Be this as it may, Dr. Christison divides the disease into three stages,—the incipient stage, which in some instances, if not in all, is a state of congestion or reaction,—the middle stage, where the cortical structure of the kidney is nearly or entirely destroyed,—and the advanced or final stage, where the tubular masses are also invaded and more or less obliterated.

1. *Incipient Stage.*—Dr. Christison admits that the organic alterations of structure which constitute the *essence* of granular degeneration, are not easily traced in the early stage. There are scarcely any symptoms to attract attention, and the kidneys are therefore seldom inspected in this stage. But Dr. Christison thinks it *probable* that the lesion is at its commencement simply a minor degree of the pathological characters of the middle stage, namely the deposition of a grayish-yellow, obscurely-granular matter in the cortical structure, with, or possibly without, some degree of sanguineous congestion.

"In some cases however, though comparatively few in number, the disease commences more with the characters of an acute affection. And then it may prove fatal soon after its appearance, either simply from the immediate effects of the disturbance to the renal functions, or from the development of acute, secondary disorders. Several instances of the kind have now been published by the different authors who have made the subject a study; and I am enabled to add one more to the list. From an examination of the facts hitherto collected it appears, that where the disease in its acute form proves fatal in the early stage, the kidnies are found flabby, friable and unusually large, sometimes more than twice the natural size; much darker and more vascular externally, and with points and star-like spots of ecchymosis; internally dark, brownish-red, or almost reddish-black, gorged more or less with blood, which drops from a cut surface in unusual quantity; and they often present throughout their whole structure, but more especially in their cortical portion, lines, small roundish specks, or stellated spots of still darker colour, like ecchymosis, and not easily removed by washing. *Rayer* thinks he has ascertained that these spots are often the Malpighian glands in a state of congestion. Sometimes this congested state of the kidney prevails throughout the whole organ equally. At other times, as in the case recorded in the Appendix (Case 1.) the cortical structure seems chiefly affected, and presents a more distinct and more coarsely striated appearance than natural, probably from blood being injected or extravasated in lines into the fundamental cellular tissue. The cortical structure almost always seems considerably broader than in the healthy state, as if it were distended towards the circumference by its gorged condition,—a state which the French pathological writers consider to be hypertrophy of the organ. Occasionally there is also an appearance in the cortical substance of a granular matter of a dark reddish-yellow tint deposited here and there; so that its natural striated texture is somewhat obscured. But the dark colour of the parts renders it very difficult to determine these points accurately. Case 1. however seems a clear example of the kind now referred to.—The lining membrane of the pelvis of the kidney is commonly very vascular and red." 6.

The bladder is always found much contracted, and commonly contains only a drachm or two of pale, coagulable urine, varying in density from 1014 to 1025. When death ensues from coma, this has nothing to do with extravasation, or congestion, or serous effusion. Sometimes, however, continues Dr. C. there is found extravasation on the surface of the brain, thickening of the pia mater, effusion of serosity in the subarachnoid cellular tissue, or into the ventricles, congestion of the vessels of the membranes or brain, darkness and injection of the cineritious substance, morbid tumors, &c.—some one or another in short of those organic changes which are wont to be found after death from apoplexy. These appearances, more especially congestion and serous effusion, have been far from uncommon in the practice of Dr. Bright. *M. Sabatier* mentions that effusion of serosity is common enough among children. Dr. C. adds that, in Edinburgh, they are certainly much less frequent, and the generality of cases of death by coma present no morbid appearances of any kind within the head.

So far as we have seen, coma from diseased kidney has been attended with either congestion or serous effusion within the head. It would be difficult, we conceive, for any candid pathologist to believe that renal coma is *usually* unattended with encephalic alterations. Nor is it likely that it would be so. If the retention of urea in the blood occasions coma, it is à priori likely that it would occasion it as most other substances do, by affecting the vascular system of the brain. It must, at the same time, be admitted that the fact mentioned by Dr. Christison is an important one—we allude to the infrequency of cerebral lesions in renal coma at Edinburgh.

The other secondary lesions, more particularly inflammation of the serous membranes, and dropsical effusions into the cellular tissue and the serous sacs, are rather referred and referrible to the more advanced stages of the malady.

"The blood often contains urea in large quantity; and this principle also exists in the serosity of the brain. It may be presumed to be also present in other secretions. This impregnation of the fluids with urea is an invariable fact where the urine has been suppressed or much reduced before death. It was first announced by myself in my paper in 1829; since then I have repeatedly verified the fact; and on several occasions it has been corroborated by the experience of my hospital colleagues. Those therefore who have called in question the accuracy of the original statement have either manipulated incorrectly; or they have looked for urea where it was not to be expected,—namely in cases of death from other causes besides coma, and where the urine was not defective materially in the amount of solids daily discharged." 9.

Dr. Christison attempts with diffidence, and wavers in the effort, to connect the middle stage of the disease with what has been described as the first. The link may exist but it is very indistinct, and its characters must be left to further observations and more precise inquiries. We therefore pass to the second stage, and leap over the dubious ground that separates or connects them.

2. *Middle Stage.*—Dr. Christison remarks that the deposition of granular or cheese-like matter, the only important and well-established anatomical character of the morbid formation, seems to be for the most part confined at first chiefly to the cortical structure of the kidney. There are exceptions: but they are few in number. As the morbid deposit advances, the natural structure of the organ gradually disappears; at length the former takes the place entirely of the latter; and still the tubular portion of the organ may remain little, if at all affected. This, which constitutes his middle stage, is, in the majority of cases, well defined, not only by its anatomical characters, but also by the circumstance that now the disease first manifests itself by symptoms.

"The kidney is now sometimes larger than natural, sometimes of the natural size, very rarely somewhat diminished. Its consistence varies: If enlarged it is commonly softer than in the healthy stage, at times even friable: If diminished, it is on the contrary for the most part rather firmer, at least of natural firmness. Its colour externally is paler, sometimes uniformly grayish, grayish-yellow, or yellowish-red, more commonly of its usual brown tint, but of a paler shade, minutely mottled with gray or yellowish-gray, and often traversed by white indurated streaks, like cicatrices. When its investing membrane is removed, which for the most part may be done with facility, the outside of the kidney is seen to be more distinctly mottled brown and gray, or else uniformly grayish or yellowish, with numerous spots of vascularity, often forming lines or stellated chequerings. The surface has consequently a granular appearance, and is often actually rough from a distinct granular structure. But in this stage there is seldom the lobulated division, and very rarely the botryoidal surface, which frequently occur at a more advanced period. When a longitudinal section is made through the kidney so as to divide it into two symmetrical parts, the external portion usually occupied by the cortical structure is seen broader than natural, sometimes of the natural breadth, sometimes much narrower; and its breadth appears to depend upon whether the kidney is enlarged or contracted. This part, instead of presenting its usual reddish-brown colour, and the appearance of coarse striæ in nearly parallel lines, lying in a direction from the centre of the organ towards its surface, is grayish, grayish-red, grayish-yellow, or reddish-yellow, without any striated arrangement or parallel lines, but of a uniform, sometimes obscurely, sometimes distinctly, granular texture, chequered occasionally with reddish or brownish spots. And there is no distinction, no boundary line, between this structure and that which dips inwards between the tubular masses. When the kidney is injected, the matter according to *Dr. Bright* does not penetrate into the cortical portion. The granular structure of the morbid formation is sometimes quite distinct; more generally it is obscure to the naked eye on a cut surface, but becomes visible with a common magnifier or upon tearing the morbid tissue; but occasionally neither by tearing, nor with the aid of a magnifier of four or five powers is the granular character to be distinctly seen, or any other than a smooth homogeneous structure like that of the brain; and in a few cases the structure is not only homogeneous, but friable, and not unlike the fatty degeneration of the liver, although there is no fatty matter in it. Hence some with reason call in question the propriety of the term *granular degeneration* as applicable to all these forms; while others with no less reason suspect that the several forms may be connected with morbid deposites positively different from each other in nature." 15.

The cortical portion of the kidney may even be completely disorganized, without the tubular part being distinctly altered. But the morbid deposit may extend between the tubular masses, or even among the rays at their bases, before the disappearance of the striæ of the cortical portion. When the tubuli are thus invaded, their striæ are finer and less distinct than natural. Sometimes, according to Rayer, they present red indurations of their papillæ.

If death occurs in this stage by coma, with diminished urinary secretion, the bladder is found contracted; and the urine contained in it has a density for the most part between 1010 and 1016, and coagulates more or less with heat and nitric acid. Urea is invariably found in the blood as well as in the serous fluids effused in various quarters. There is rather less appearance than usual of vascularity or injection of vessels in the various membranous textures of the body, as well as in the brain. So, at least, it has been with Dr. Christison.

At times, no organ save the kidney is diseased. But often various morbid alterations have been silently proceeding. The most frequent secondary appearances, continues Dr. Christison, are dropsical effusions, namely œdema of the cellular tissue at large, œdema of the lungs, serous effusion into the sacs of the peritoneum, pleura, and pericardium,—emphysema of the lungs, with redness of the bronchial membrane and mucous gorging of the bronchial tubes, as the consequences of catarrh,—the signs of recent inflammation of the lungs, redness, serous or sanguinolent infiltration, or hepatization of their tissue—the signs of recent pleurisy, peritonitis, or pericarditis, such as turbid serum effused into the cavities, and soft, curd-like lymph upon the membranes,—traces of inflammation of the alimentary canal, more especially of the intestines, redness of the mucous membrane, effusion of lymph on it, enlargement of its glands, or ulceration,—tubercular deposition in the liver,—softening of the spleen,—hypertrophy and dilatation of the heart, sometimes with valvular obstructions, sometimes without them,—enlarged mesenteric glands. Among the rarer appearances may be mentioned œdema of the glottis, ulceration of the larynx, redness of the villous coat of the stomach, vascularity of the mucous membrane of the urinary bladder, induration of the spleen. In many cases too, traces are found of old attacks of visceral disease, more especially of pneumonia, pleurisy, peritonitis, and pericarditis. These secondary derangements sometimes occur in the incipient stage, especially those indicating recent inflammation. They are much more frequent however in the middle stage, and still more so when the primary disease has made farther progress.

3. *Advanced Stage.*—The morbid deposition makes progress, and affects the tubular, as it did the cortical portion of the kidney. At first, the grayish yellow matter is partially deposited between the tubular masses or even among the fibres of the tubuli,—in the one case appearing to flatten the tubuli, and in the other to expand their bases. As the disease advances, the kidneys usually diminish in size, occasionally to such a degree as not to exceed two inches in length.

"Their surface is sometimes lobulated, commonly pale, very frequently rough and irregular, or even botryoidal, like the roe of the salmon, or the mineral pisolite. The common colour outwardly is uniform pale grayish-yellow, sometimes with vascular spots, more commonly without them; but when very much reduced in size, the proper brownish-red tint is often preserved. Their firmness varies exceedingly, from that of healthy liver to that of the same organ when affected with hard tubercle; and *Dr. Bright* has sometimes found them so hard as to cut almost like cartilage. A longitudinal section displays various appearances, according as the kidney is diminished or not in size. If it is not less than natural, the portion usually occupied by the cortical structure is of the natural breadth and entirely occupied by grayish-yellow granulation, or by a homogeneous substance somewhat like fatty degeneration of the liver; the same matter is extensively deposited in the central portion between the tubular masses, and frequently among the striæ of the tubuli themselves; and the tubuli are pale flesh-red in colour, more finely striated than natural, compressed, diminished, broken up, or some of them even entirely obliterated, and their place occupied by the morbid deposit. If the kidney, as more generally happens, is reduced in size, the cortical portion is contracted in breadth ; so that the outer extremities of the tubular masses are pushed as it were towards the external surface. The tubular portion presents the same appearance with what has just been described ; but the granular deposit among the tubuli is less extensive. In the progress of matters the kidney is sometimes seen converted into one entire mass of uniform granular or homogeneous degeneration, with the exception of a single tubulus at one end, or perhaps one at each extremity. In other instances, where the morbid deposition has been either scanty from the first, or has subsequently been absorbed, we sometimes find one of the kidneys exceedingly small, flabby, thin, and so entirely deprived of its proper structure, that no vestige of either cortical or tubular substance remains. In such cases, the ureter is of course useless ; and in one instance of the kind *M. Solon* found its canal obliterated ; but in all which have come under my observation it was pervious. In a few instances firm tubercle-like masses are diffused throughout the softer granular matter. More frequently there are little cavities or cysts interspersed ; which are either true cysts, or more commonly the infundibula remaining after their corresponding tubuli have been destroyed. It is usual to find one kidney more advanced in disorganization than the other; and in general this is the right. Occasionally one of them is in the very last stage of the disease, the whole cortical and tubular structure having disappeared, while the other is but slightly advanced in the final stage, or even in the middle stage only. By thus comparing the state of the kidnies in the same cases, the successive stages of the disorganizing agent are traced, where otherwise it might be very difficult to suppose that the appearances are nothing else but different steps of one organic derangement. We may thus for example feel in some measure assured, that excessive contraction or atrophy of the kidney, whether with or without hardening of its substance, may be the effect of granular degeneration, and not, as *M. Rayer* seems to think, of simple chronic nephritis only." 21.

Rayer and Dr. Osborne have remarked that the renal veins often present firm fibrinous clots, ramified into their branches, and occasionally adherent. The supra-renal glands are commonly enlarged and indurated in the advanced stage. Sometimes even the fat around the kidneys acquires a firm consistence and somewhat granular structure.

There is a little blood in the heart and great vessels. Indeed the whole body presents all the appearances of excessive hæmorrhage. If many organs are usually diseased when death occurs in the second stage, à fortiori we shall find them so after death in the third. A common combination of lesions is granular derangement of the kidneys, tubercular liver, and hypertrophy of the heart, in addition to which there is not unfrequently, either emphysema of the lungs with catarrhal effusion, or recent inflammation of some serous membrane.

The chemical constitution of the matter deposited in granular degeneration of the kidneys has not yet been determined.

### SYMPTOMS AND HISTORY.

The symptoms must be viewed as indicative of the primary disorder, and of the secondary affections to which it gives rise. For the sake of precision and distinctness, it is necessary to separate these two classes.

Though granular disorganization of the kidney is usually at last a chronic disease, it may commence in either an acute or chronic form.

*Acute Form.*—This usually begins with decided symptoms. Generally, after exposure to cold, there is chilliness or a rigor followed by pyrexia. At the same time the urine becomes quickly scanty, at times almost, nay altogether, suppressed, highly albuminous, occasionally bloody, and in rare instances mingled with clots of blood. There is also for the most part frequent desire to pass urine, with at times difficulty or positive pain in discharging it,—not uncommonly dull, more rarely acute, pain in the loins, aggravated by pressure, and sometimes shooting downwards to the inside of the thighs or external parts of generation,—and more usually pain across the pit of the stomach, and in the flanks, either felt only on pressure, or increased by it, but constantly present more or less. Sickness and vomiting are of common occurrence. This train of symptoms does not continue long, seldom indeed above two days, without the supervention of others, belonging to the secondary affections. Of such the most common are dropsy, especially of the limbs and face,—coma, with or without convulsions,—and acute serous inflammations, more especially pleurisy. Above all, however, dropsical effusion, in one shape or another, is seldom long absent; and not unfrequently it puts on the characters of what has been termed "inflammatory dropsy."

The disease may be checked by active treatment; or coma, or some acute serous affection may carry off the patient, even in four or five days. Most commonly, the symptoms lapse into those of the chronic stage. Complete recovery may undoubtedly take place; but it is often only temporary, and, in other instances, it is merely apparent, because permanent organic injury has been done to the kidneys, and the urine continues essentially morbid, although for a long time there may be no other sign of a deviation from a sound state of health.

Acute granular degeneration does not always present the characteristic symptoms, which have been enumerated. It is more common to find dropsy or some other secondary affection developed soon after an attack of rigor, without any other interposed or concomitant symptom except scantiness and an albuminous impregnation of the urine. The real disease may thus, and not unfrequently does, escape notice altogether.

In other instances, the disease appears to begin in the acute form, but has actually existed unnoticed in the chronic, for months.

*Chronic Form.*—This may be merely the sequela of the acute form. More frequently the disease is, from the first, insidious in character, and slow in progress. The patient's attention may not be called to it, until it has advanced considerably.

"Nevertheless even in such cases an attentive examination will not unfrequently show, that warnings of mischief going on were not altogether wanting, though the patient had neglected them. Thus it is not uncommon to find in cases apparently the most obscure in their origin, that the urine has been very long scanty, or on the other hand too abundant, or occasionally of a cherry-red colour from a little blood,—or that it was passed frequently and with difficulty, or with positive pain,—or that there were frequent gnawing pains in the loins or flanks, extending at times to the thighs or groins or scrotum. No single symptom of the kind now alluded to has appeared to me so invariable, or of so much service for indicating the commencement of the disease, as the fact of the patient being regularly awakened once or oftener in the night time by the necessity of passing urine. I have scarcely ever known it wanting, where any other local symptom existed; frequently has it been present without any other for a great length of time; and it is so remarkable a deviation from the ordinary rule of health, that, although it may have been neglected, no individual can fail to recal it when his memory is tasked on the subject by his physician." 20.

We cannot say that we attach the same degree of importance to this symptom, as Dr. Christison appears to do. It is usually present in degeneration of the kidney; that we grant. But it is also present in many other cases independent of renal disorganization. It is constantly observed in cases of "irritable bladder," and passes away with its other symptoms, perhaps never to return.

Dr. Christison goes on to observe, that the period during which the malady remains obscure is altogether uncertain. It may be one of months or of years.

"While matters remain in this position, incidental causes may suddenly develop the acuter symptoms detailed above; and more frequently the like causes give rise to some secondary disorder. The essential disease however is distinguished by the following indications. There is reduction of the strength; emaciation, not always, however, considerable; a remarkable uniformity, and commonly great paleness, yet on the other hand at times a peculiar pale-brownish dinginess, of the complexion; defective transpiration as indicated by a dry state of the skin, and the want of perspiration under exercise; often a tendency to drowsiness; often too weakness of digestion or even well-marked dyspepsia, not unfrequently attended with sickness or retching in the morning on awaking from sleep; thirst;—together with an important pathological condition both of the urine and of the blood, and sometimes one or more of the various annoyances already specified as apt to attend the discharge of urine. Of these symptoms none are invariable except the altered state of the urine and blood, with perhaps also the unhealthy complexion. The two former are not only invariable, but likewise very characteristic, and therefore of great importance; since they are alone amply sufficient to point out the true nature of the case, and will also in my opinion fix with considerable precision the state of progress of the disease. They will therefore require some detailed consideration." 32.

*State of the Urine.*—To this Dr. Christison passes, and, as he is well qualified to speak upon the subject, we shall not treat his observations lightly.

In the early stage of the acute form, the urine is sometimes natural in quantity, very rarely increased, far more generally diminished. Instead of passing between thirty-five and fifty ounces a-day, which constitute the average range of health, the patient discharges only eight, twelve, or sixteen ounces; sometimes the quantity does not exceed two or three ounces; and occasionally the secretion is altogether suppressed. The latter circumstance is the harbinger of coma.

"The urine is also much altered in its constitution. Sometimes it presents a blood-red tint of more or less intensity: occasionally the tint is so deep as to render it opake ; in a few instances clots are intermingled with it ; and still more rarely the fluid discharged seems to consist of nothing but blood, which afterwards partially coagulates.* Most frequently of all, however, the colour of the urine deviates little from that of health ; but in that case it is often rendered muddy or slightly opalescent by the presence of fine light particles, which do not disappear on the application of gentle heat, and consist probably of one of the modifications of the mucus of the bladder pointed out by Berzelius.—A sediment too sometimes forms when the secretion cools, which is most generally lithic acid or the lithate of ammonia, and which is re-dissolved at a gentle heat, lower than what is required for the coagulation of albumen. Occasionally I have observed a white sediment of the earthy phosphates ; but this is exceedingly rare except as the result of alkalinity of the urine induced by long standing and consequent decay.—In general the urine froths more than usual when shaken ; and on blowing into it through a tube bubbles are formed as with soapy water. This property is confined however to the urines which are loaded with albumen.—Many specimens of urine in this stage are much more prone to decay than the healthy secretion. In some I have observed a decided ammoniacal adour so soon after its discharge that in all probability decay must have commenced within the body ; and frequently so much carbonate of ammonia is formed in eight or ten hours that a powerful ammoniacal odour is exhaled, earthy phosphates are thrown down in abundance, brisk effervescence is caused by acids, and another character to be stated presently, coagulation by heat, may be prevented from being developed.—The density of the urine does not differ much from the natural standard." 34.

To determine this, the first urine voided by the patient in the morning should be made the subject of examination. Otherwise it will be modified by the influence of diuretics, drink, cold, and so-forth. But the most remarkable property which the urine exhibits in this stage is its coagulability. Dr. Christison owns and enumerates many sources of this as a temporary occurrence, and he therefore admits that it is incorrect to hold, as some do, that albuminous urine is pathognomonic, or by itself characteristic, of granular disorganization of the kidneys. At the same time he is convinced that there is no other cause, or rather there are no other causes taken together, by which an albuminous impregnation is so often induced as by the disorder in question. He is persuaded likewise that no other cause whatever will occasion the enormous accumulation of albumen in the urine almost invariably observed in the early stage of the disease where it commences with acute symptoms.

Speaking of the tests, and arriving at the conclusion that heat and nitric acid are the best, Dr. Christison adds these cautions.

"It is advisable to make use always of both tests; and this for several reasons. For first, if the urine is ammoniacal, the action of heat may be prevented even where the proportion of albumen is great. Secondly, heat alone may occasion a flaky precipitate where there is no albumen, owing to the superabundance and consequent separation of earthy phosphates,—a deposition, which nitric acid will both prevent and remove. And thirdly, nitric acid alone may

---

* "Care must be taken, as Mr. Rees has suggested, not to mistake for the effects of disease the colours imparted to urine by many articles of vegetable food. Hæmaturia from other causes may be distinguished, as stated in M. Solon's work, by the urine ceasing to present coagulability so soon as it ceases to be red."

occasion a flaky precipitate of lithic acid; which however is re-dissolved by an elevation of temperature, while albumen remains insoluble. In regard to these sources of fallacy, I have to observe in the first place, that the urine should be always tested if possible before it decays and becomes ammoniacal. Because I have found that sometimes even nitric acid added in excess did not separate albumen which had been present in large quantity,—a fact which is probably to be ascribed to the albumen having itself undergone more or less decay along with the other principles of the urine." 44.

The quantity of albumen, though various, is always abundant in the early stage. Ten parts by weight in a thousand of urine will render it almost a thin uniform pulp when heated. Less is seldom met with in the early stage. The highest Dr. C. has yet found has been twenty-seven parts in a thousand. Here, as in all similar instances, heat converted the urine into a gelatinous mass, from which no fluid issued on turning the tube upside down.

It is remarkable, he continues, that in some instances the albumen suddenly and for a time disappears from the urine. This occurrence is more frequent in the more advanced stages of the disease. But Dr. C. has seen it at the commencement of the complaint.

Dr. Christison disputes the idea of some that the albumen is a substitute for the urea, which is deficient. He believes that the only explanation of its occurrence must be found in some peculiar irritation of the kidneys.

The urine contains an unusually small quantity of solid ingredients. This character, Dr. C. goes on to state, though presented more or less in every stage of the disease, is commonly much better marked when it is somewhat advanced, than at the commencement. But it is also in general a well-defined character from the beginning, provided attention be paid to two conditions,—first, that the natural course of things shall not have been disturbed by treatment,—and secondly, that the quantity of solid ingredients discharged by the excretion be taken absolutely, not relatively to its watery part. Even the proportion of solids relatively to the fluid part is very generally somewhat diminished; because, although the density of the urine may be so high as 1020, or even 1024, this is partly owing to the presence of the foreign ingredient albumen, and consequently when the fluid is filtered after coagulation the density falls by four, five, or even seven units. But if an account be also taken of the defect in the quantity of urine, then the absolute amount of solid ingredients excreted in the twenty-four hours is almost always, perhaps invariably, very deficient. Twelve ounces of urine, of the density of 1016 after separation of the albumen, will probably constitute a fair estimate of the daily discharge in a very great proportion of cases at a moderately early stage; and this estimate infers the diminution of the daily discharge of solid matter to at least one fourth or nearer one-sixth of the healthy-average. Among the other solid ingredients, the urea is affected to a full extent.

On the whole, Dr. Christison sums up the pathognomonic characters of the urine in the *incipient* stage of granular degeneration of the kidneys, as— a reduction, but only a moderate reduction, of density, a strong albuminous impregnation, and a material diminution of the daily discharge of solid ingredients.

Passing over some remarks on the colour of the urine, and merely pausing to observe that strings of vesical mucus are sometimes seen in it, we proceed to notice that, as the granular deposition proceeds, the density sinks from the standard formerly mentioned to 1016, 1014, 1012 ; and when the case has reached the advanced stage it is usually so low as 1010, 1008, or 1007, even where the quantity is rather under than over the natural standard. The lowest density Dr. C. has ever accurately noted was 1004. The amount of albumen now varies much more than in the early stage. When the proportion is as great as in that stage, above all if the density be exceedingly low, it will be found that the patient labours under symptoms of reaction or local inflammation, and that the acute form of the disease has been casually reinduced. More frequently the albumen disappears nearly or entirely, either for a time or altogether. This happens much more commonly in the advanced than in the early stage ; when the disorganization of the kidneys has proceeded far, slight haziness is all that heat or acids will occasion ; and often enough no alteration whatever is produced. Even in such circumstances however, the incidental recurrence of acute symptoms, attended by increased irritation of the kidneys like that which occurs often at the commencement, may cause the albumen to reappear in abundance for a time. But Dr. Christison esteems it a great mistake to suppose that the quantity of the albumen necessarily increases with the increase of the disease. The very opposite is the general rule.

Another invariable feature of the urine in the advanced stage is a diminution of its solid contents, both relative and absolute.

"The total solids of the urine in a state of health I have found to be 67.7 parts in one thousand, when the density is at 1029, and the quantity thirty-four ounces avoirdupois. When the disease was far advanced, the proportion of solids has been 24 parts in one thousand, where the quantity of urine amounted to twelve ounces, and the density was 1009.5 ; and the lowest proportion I have ascertained by actual experiment was 15 parts in the thousand where about thirty-six ounces of urine were passed of the very low density of 1006.9. In the latter case the total solids discharged throughout the day were reduced to one-fifth of the healthy average ; and in the former to nearly one-twelfth.—It is not improbable that this great reduction may affect certain ingredients more than others. A few experiments seem to lead to the conclusion that the lithic acid and salts of the urine are more diminished at times than the urea. But this is by no means an invariable fact ; neither has the difference ever appeared to me very material ; so that upon the whole it cannot be included, without farther investigation, among the morbid characters of the urine." 55.

The pathognomonic characters of the advanced stage are summed up as a great reduction in density, and an equal reduction of the daily discharge of solids,—frequently associated with the presence of albumen in small quantity. Deviations are however more apt to occur here than in the early stage ; and among these the most important to be kept in view are the frequent absence of albumen, and the daily discharge of the full amount of solids owing to spontaneous diuresis. The only character absolutely invariable is great lowness of density, with of course a reduced *proportion* of solids.

Dr. Christison protests against the notion that the presence of granular degeneration of the kidney may be known by the condition of the urine alone.

"It is partly to correct such notions that I have dwelt so fully on the various properties of the secretion at different stages. Any one who peruses attentively what has been said will perceive, that it must be sometimes impossible to rest the diagnosis on the state of the urine alone. In general indeed we may do so with confidence. For *first*, when the disease has continued for a short time with acute symptoms, the characters of the urine, namely a somewhat reduced density, a diminished amount of daily discharge of solids, and high coagulability are invariable, and do not occur conjunctly so far as is yet known in any other disorder. *Secondly*, there is a very common conjunction of characters in the advanced stage, which has seemed to me never to occur in any other malady, namely great reduction of density, some diminution of quantity, much diminution of the daily discharge of solids, and slight coagulability. *Thirdly*, another conjunction not less characteristic perhaps is great reduction of density, slight coagulability and a great increase in quantity, consequently with little or no diminution of the daily discharge of solids. *Fourthly*, I have never in any circumstance, except in the advanced stage of granular disorganization of the kidneys, met with urine about the natural standard in quantity, of the very low density of 1006 or 1008, consequently defective materially in the daily discharge of solids, almost colourless, or cherry-red, or smoke-brown, or orange-yellow, and obscured by opalescent muddiness which does not disappear under rest or gentle heat,—even although not coagulable. *Fifthly*, though not absolutely prepared to state the same proposition where the quantity of urine is superabundant, and its other qualities such as those last described, I am inclined to think this condition also characteristic. But there is another condition met with occasionally which is common to this with other diseases,—namely where the quantity is natural or abundant, the density low, the daily discharge of solids natural or under the healthy standard, the colour pale-straw yellow, without either coagulability or permanent muddiness. This is the state of the urine which we observe in many diseases, and which even seems natural to some persons in good health. It is also however compatible with the presence of granular disorganization of the kidneys ; and it has seemed to me to occur in one of two remarkable circumstances,—either when the disease is checked in the early stage, and a cure is going forward,—or when the disease is in the advanced stage, but has arrived at a temporary suspension of its progress." 57.

Dr. C. passes, and we pass with him, to the :—

*State of the Blood.*—We cordially agree with him that much may be hoped and expected from the progress of analytical chemistry, and its application to pathology. We are now almost sufficiently acquainted with the coarser and more palpable alterations of the solids—those of the fluids, and more particularly of the blood, are an almost untrodden field. Dr. Christison apologises for the imperfect character of his own observations, and remarks, with justice, the difficulty of fixing the real value of any, from the presence of a sufficient number of comparative analyses of the blood in other diseases. He presents, however, the facts that he has ascertained, and offers them as contributions to a future stock.

In the early stage, when the symptoms put on the acute form, the blood generally has the characters of inflammatory action. It is commonly cupped and buffed, and the serum is usually somewhat lactescent, and yields to sulphuric ether when agitated with it a small quantity of concrete oily matter, which seems to differ little from the fat of the cellular tissue.

" The most remarkable alteration however of the serum, is a great decrease in density, together with a corresponding reduction of its solid contents. This state of the serum, which was first noticed by *Dr. Bostock* in some experiments made at the request of *Dr. Bright*, and which was afterwards observed also by myself in 1829, and by *Dr. Gregory* in 1831, has appeared to me an invariable character in the early stage. And it farther seems to be with certain exceptions peculiar to that stage. The amount of reduction varies in different cases. It is always however very considerable,—the density which ranges naturally between 1029 and 1031, being seldom above 1022, often so low as 1020, and occasionally even 1019, [Case 7]—and the solid contents being reduced, from 100 or 102 in one thousand, to 68, 64, or even 61. The reduction, so far as I may judge from some not very careful trials, would seem to affect equally the albuminous and the saline contents. It occurs only when there is an abundant discharge of albumen with the urine, but then invariably. On account of the loss of albumen the serum coagulates loosely when heated." 61.

Another characteristic peculiarity of the serum, is the presence in it of a large quantity of urea. On this point, Dr. Christison speaks with the confidence of positive experiments. Urea, he states, is invariably found in the serum at all stages of the disease when the daily discharge of it by the urine is diminished materially, that is to about one-third of the natural amount. Hence it may be usually discovered in the early stage of the disease, provided the quantity of urine have not been considerably increased by incidental causes beyond what constitutes the common average at this period. But if the urine approach the healthy standard in point of quantity, and still more if it exceed that amount, urea cannot be detected satisfactorily, although still traces of its existence may be elicited. The most certain method of separating it is to evaporate the serum to perfect dryness in the vapour-bath, to boil the pulverized residue in absolute alcohol of 796, to drive off the alcohol, to dissolve the residue in water, which must be afterwards filtered through a previously moistened filter for the separation of fatty matter, and lastly to concentrate the watery solution to a small bulk, and add half its volume of nitric acid in a watch-glass. Sometimes immediately the whole mass becomes solid as it were, by the abundant crystallization of nitrate of urea; sometimes a scantier crystallization forms in a few minutes or an hour at farthest; and occasionally the only indication of the presence of urea is some effervescence, attended with the peculiar odour which accompanies the action of the acid upon this animal principle as it exists in urine. Two or three hundred grains of serum are commonly sufficient for analysis, where the urea is not in very small proportion.

The proportion of fibrin is usually increased in the early stage, in the ratio of the inflammatory action present, and of the buffy condition of the blood. He thinks that the proportion of hæmatosin is little, if at all affected. We are speaking still of the early stage.

From these statements, into which we have not gone quite piecemeal, it follows, as Dr. Christison himself sums up, that the condition of the blood in the early stage of the disease is characterized by the low density of its serum and a defective proportion of albumen, by the frequent presence of urea, by the frequent increase of the fibrin, and by the proportion of the hæmatosin being unaffected. And in order to test its characters in these respects with accuracy, the precautions to be kept in view are that the disease shall be really recent, not preceded, as the acute symptoms often are, by latent disorganization,—that there shall have been no marked state of precursory ill-health from other causes,—that in particular blood-letting shall not have been practised recently before,—that the urine shall be under the natural standard in point of quantity,—and that symptoms of re-action shall be present. The last two conditions are the circumstances which regulate the presence of urea, and the increase of fibrin.

As granular disorganization advances, important changes in the blood occur. The serum is commonly not so lactescent. The clot less frequently presents the buffy coat; yet, if there be incidental inflammatory action, this appearance shews itself, and indeed it *may* do so, independently of that occurrence; when there is a buffy coat the clot is contracted and small.

"In the next place the density and solid contents of the serum, which have been shown above to be invariably much reduced in the beginning of the disease; gradually return to the healthy standard, or even exceed it. In the middle stage the serum may be often met with of rather low density, such as 1025 or 1024; and this state is always found to concur with considerable coagulability of the urine. Sometimes too, even in the most advanced stage, the density is reduced as low as it ever is at the commencement, provided accidental reaction occur and thus render the urine highly coagulable. But the ordinary course is for the density and solid contents to be restored as the disease advances; and this restoration keeps pace with the gradual diminution and disappearance of albumen from the urine. In the middle stage, when the density is about 1024, the proportion of albumen and salts of the serum amounts to 630 or 660 in ten thousand parts of blood. In the most advanced stage where there was no reaction and very little coagulability of the urine, I have seen the density of the serum 1031, and the proportion of its salts and albumen to the entire blood so high as 973 in ten thousand parts. This is above the healthy standard, which according to Lecanu varies between 780 and 800,—and according to my own experiments between 816 and 853. In the same stage, in a case where general reaction and pleurisy had supervened, the density of the serum was 1021, and the solids of the serum amounted only to 583 parts in ten thousand of the blood.—In the third place, the urea frequently disappears from the serum of the blood as the disease advances; but in the most advanced stage it commonly reappears, and it is sometimes present towards the close in larger proportion than ever. The cause of these variations is apparent. The urine in the middle stage, though defective in the proportion of solid ingredients, is often not so in the total amount of them discharged daily; because, though low in density, it is frequently increased in quantity: But as the disease draws towards a close, the quantity decreases as well as the density; and at length sometimes there is an almost total suppression. Here in short, as in the early stage, wherever there is a material reduction of the daily discharge of urea, it may be distinctly found in the blood; but not otherwise." 69.

*In the fourth place*, the fibrin is most commonly natural in proportion, after the early stage is passed, unless inflammatory action occurs, when it is of course, increased. *Lastly*, by far the most remarkable character of the blood, in the advanced stage of the disease, is the rapid diminution of its hæmatosin. This may sink, so as to form less than a third of the healthy average. The effect of the disease in this respect is apt to be complicated with that of occasional blood-letting, which has a very great influence in reducing for a considerable length of time the proportion of colouring matter in the blood. But the reduction which takes place in granular disorganization of the kidneys is far beyond what can be accounted for by the extent to which blood-letting has been usually carried; and besides is found to be excessive where no blood has been lost at all previous to that which is analysed.

"I am acquainted with no natural disease, at least of a chronic nature, which so closely approaches hæmorrhage in its power of impoverishing the red particles of the blood. It was stated above that the average proportion for the male sex is 1335 parts in 10,000; and that in the first week of the disease I had found it to be 1339 in a stout man not previously bled. In another man also of stout habit, one month ill, but once or twice previously bled, it was 1111; in another powerful man five weeks ill, and once before slightly bled, it was 1046 [Case 2;] in a stout porter, ill probably for two months, and once before moderately bled, it was 955; in a lad two months ill, and bled once before, and that recently and largely, it was 564 [Case 7;] in a gentleman six months ill, and not bled for eighteen months, when he had a severe attack of pneumonia, it was 491 [Case 14;] and in a young man ill for three months and a-half subsequent to scarlatina, and who had never been bled before, in was only 427 [Case 10.]" 72.

Thus in the advanced stage of this formidable malady, the proportion of hæmatosin is invariably and greatly reduced; frequently the solids of the serum are also defective, sometimes on the contrary in excess; and not unfrequently, especially if the disorder is very far advanced, the serum likewise contains urea.

The leuco-phlegmatic complexion is one of those symptoms which flows directly and obviously from the reduction of the quantity of hæmatosin. Sometimes a pale, transparent, waxy, and very characteristic hue is produced. At other times, says Dr. Christison, a peculiar dingy brownish tint is communicated, which seems to depend in general on the original complexion being dark, but is occasionally observed also where the natural complexion had been fair and florid. The latter appearance is most distinct in those who have never had dropsical effusion along with the primary disease, or who have been much affected with disorder of the functions of the stomach; and we sometimes observe the pure leucophlegmatic tint give place to the dingy brown hue in the advanced stage in those, who had been dropsical at the beginning and subsequently got rid of the anasarcous effusion. Where the countenance is somewhat puffy from œdema and the primary disease is moderately advanced, the leucophlegmatic waxiness of the complexion is most characteristically developed. Sometimes the proper pale or dingy tint is altered, either by general reaction inducing some degree of florid flushing of the face, or more frequently by obstructions to the respiration or to the action of the heart creating an obstacle to the free return of blood by the veins, and so occasioning lividity.

### III. Secondary Diseases.

These form the subject of the third section. We shall not follow Dr. Christison so closely as we have done. The chemical and analytical ground over which we have passed is that which Dr. Christison is eminently calculated to cultivate with ability and success.

Anasarca is by no means a necessary consequence of granular degeneration. This, however, seldom continues long without additional symptoms and diseases supervening.

The secondary affections of most frequent occurrence are dropsy, diarrhœa, pleurisy and peritonitis, pneumonia, catarrh, dyspepsia, and chronic vomiting, coma with other affections of the head, chronic rheumatism, organic diseases of the heart, and organic diseases of the liver.

1. *Dropsy.*

Anasarca, as our readers know, is the most frequent of all the secondary affections. It has been viewed, indeed, as an essential symptom ; but this is certainly erroneous. According to Dr. Christison, the proportion of dropsies dependant in part or wholly upon organic disease in the kidneys is, in Edinburgh, not less than three-fourths of the whole. At Strasbourg Professor Forget has found the proportion to be one-half. Of course these statements of facts preclude all argument; but we think that we are borne out in affirming that the proportion in London is by no means so great. The number of dropsies from diseases of the circulating organs, greatly, we should say, exceed those which may fairly be laid to the kidney.

The parts most commonly affected by the anasarca are the lower limbs. But the whole external cellular tissue may be so, and that of the face very often is so. When great, it may be attended with effusion into the serous cavities, but this seldom assumes a prominent form unless peritonitis or pleurisy has contributed to its development.

Dr. Christison alludes to the division of dropsies into inflammatory and atonic. His sentiments on their respective connexions with granular degeneration are not undeserving of attention.

1. "A very great proportion," he says, "of cases of inflammatory dropsy depend on organic disease of the kidneys. By inflammatory dropsy I understand serous effusion into the cellular tissue, more or less acute, and attended with distinct febrile reaction, sometimes with acute visceral inflammation. Of dropsies of this kind I have not myself met with a single case during the last nine years, where there were not unequivocal signs of the kidneys being diseased." He grants that exceptions have been taken to such statements; but he repeats that they express the experience of himself and of his colleagues. The dropsy after scarlatina is claimed by Dr. C. for the kidneys.

" On so wide a field my own opportunities have not been such as to warrant a confident opinion. I can only say, that in every case of dropsy after scarlatina which has been under my own care, and in several others as to which I have been consulted, the kidnies were affected. But it is fair to add that I have had an opportunity of once or twice examining the urine, where dropsy was conceived by other practitioners to be threatened in consequence of one of its earliest signs appearing, namely puffiness of the under eyelids; and that the urine proved to possess its natural characters. In these instances however the dropsical affection was never more unequivocally formed afterwards; and its existence at all was therefore doubtful." 81.

We certainly are not disposed to go these lengths, though we do believe that a *large* proportion of inflammatory dropsies hinge on morbid states of the kidney.

According to Dr. Christison's observation, all cases of anasarca where the œdematous parts do not pit upon pressure are connected with granular disorganization of the kidneys. Such cases are commonly of the nature of acute or inflammatory dropsy. Yet they are not so necessarily; for he has seen repeated instances where œdema, not pitting on pressure, and of considerable extent, concurred rather with an atonic state of the circulation. But in every case that has come under his notice, since 1838, the effusion was clearly connected with diseased kidneys. In the majority of cases, however, of renal dropsy, the anasarcous swelling pits readily.

"3. All dropsies where the urine is steadily above the healthy standard in point of quantity occur in connexion with granular kidney, except in the instances of dropsy attending the advanced stage of saccharine diabetes. The fact of an inordinate flow of urine attending a hydropic state of the cellular tissue, without diuretics, is one to which little attention has been paid by pathological writers. It is nevertheless of undoubted occurrence: nay by no means unfrequent. I have never witnessed it where the dropsy seemed to depend on diseased heart, lungs, or liver, provided the kidnies were not also affected. But I have repeatedly observed it where the effusion concurred with the signs of disease in the kidnies alone. I have seen the effusion maintain its ground or even increase somewhat, where the daily discharge of pale albuminous urine, low in density, and defective in urea, amounted to five, eight, or even ten pounds for weeks together, nay in one case for several months. And it is still more extraordinary, that in not a few instances of the kind the anasarca subsists or increases, though not merely the urine is abundant, but likewise the daily discharge of solids with the urine fully equals or surpasses the natural standard. Such cases are most common, as appears to me, in the middle stage of the primary disease. Few pathological phenomena are more remarkable. Yet there is an analogous fact in the production of anasarca of the limbs on some occasions in the advanced stage of diabetes mellitus, where the daily discharge of urine, and of solids with the urine, is still greater." 83.

4. Dr. Christison is inclined to think (he hardly ventures farther) that all dropsies, where the urine, not being above the healthy standard in quantity, or being below it, is also below 1010 in density, are connected with granular disorganization of the kidneys, whether the urine be albuminous or not. Dr. Christison takes this opportunity of repeating that albuminous impregnation of the urine in dropsy is not a positive indication of the kidneys being diseased—that it is not invariably present in the early stage—and that it is inconsiderable, or may be permanently wanting, in the advanced one.

Dr. Christison just touches on the probable cause of dropsy in cases of renal disease. He leans, and perhaps he is right in doing so, to the supposition of Sabatier, that the dropsical effusion arises simply from the increased tendency to transudation caused by the tenuity of the blood.

Anasarca usually exasperates all other secondary disorders, and its removal usually relieves them. Yet Dr. Christison has seen the contrary. The dropsy is generally removeable.

### 2. *Dyspepsia and Chronic Vomiting.*

Dyspepsia, which at times assumes the form of chronic vomiting, is almost on a par in point of frequency with anasarca. Dyspepsia, indeed, is common in all diseases of the kidney, but it is more particularly so in this. The affection of the stomach is most frequent and most severe in the middle and final stages of the disease. It seems to be independent of general reaction or active local inflammation, and is indeed often greatest when the circulation is unusually languid, the morbid action in the kidney purely chronic, and all other organs free of acute disease. It is, for the most part, removed with difficulty.

### 3. *Diarrhœa.*

A very common secondary affection, at least in the practice of Dr. Christison. It seems, he says, in general connected merely with inordinate irritability and increased discharge of mucous secretion; but sometimes too it plainly originates in dysentery, and is connected with ulceration of the inner membrane of the intestines. It is frequently, yet not always, attended with pain in the bowels. The evacuations present considerable variety, having sometimes the apppearance of the discharges occasioned by saline laxatives, sometimes again that of a turbid watery fluid with little intermixture of fæculent matter, sometimes that of broken-down fæces mixed with many small membrane-like shreds, such as occur in the middle stage of dysentery; and at other times blood is mingled with them in more or less abundance. This is sometimes a mild affection; more generally it is severe and troublesome; and often it is excessively frequent and exhausting. He has seen, but rarely seen, a watery diarrhœa carry off the dropsy and then cease. Diarrhœa being much more frequent in Edinburgh than in England, Dr. Christison attributes its frequency, and probably with justice, to the watery, vegetable, and frequently acid diet of the lower orders of Edinburgh.

It is most apt, he informs us, to prevail in the advanced stages of the disease. In the early stage it is not frequent and readily recedes; and brisk cathartics may be freely used without much chance of inducing a permanent diarrhœa. But when disorganization is somewhat advanced, active hydragogue cathartics are apt to bring on obstinate looseness of the bowels; and the same complaint often breaks out without any apparent exciting cause. It is commonly, and in the advanced stage of granular disorganization almost always a very obstinate affection; sometimes no remedial measures will do more than mitigate its severity; and in not a few cases it has seemed the immediate cause of death.

### 4. *Pleurisy and Peritonitis.*

These, not unfrequent secondary affections in London, are comparatively rare in Edinburgh, and, according to M. Solon, are not very often met with in France. Pleurisy is more common than peritonitis, or than pericarditis. It is often latent. Though obstinate and severe, it usually yields to active treatment.

### 5. *Catarrh.*

Dr. Christison esteems acute and chronic catarrh, especially the latter, often associated with confirmed pulmonary emphysema, often independent of it, as very frequent secondary affections. The complaint sometimes commences as acute bronchitis, but ordinarily, in hospital patients, it has presented the characters of chronic catarrh; at admission, and, so far as the histories could be trusted, it seemed to have begun obscurely and advanced by degrees, without symptomatic fever. It has has always been most severe when associated with emphysema of the lungs. In such instances the symptoms have often been very urgent, the dyspnœa being severe, the expectoration profuse, the respiratory murmur strongly catarrhal, and the gorging of the lungs well marked by dulness of percussion and absence or obscurity of respiration in the lower regions of the chest, more especially behind. It is not an uncommon cause of death where granular disease of the kidneys exists, particularly in a rather advanced stage; and it is very generally an obstinate affection. Nevertheless it is often removed by proper treatment. On the whole, however, when severe catarrh, whether acute or chronic, frequently recurs or assumes an obstinate character, the complication is unfavourable, and the patient seldom survives long, though the immediate cause of death may be some different disorder.

### 6. *Coma and Apoplexy.*

Affections of the head more or less allied to apoplexy, appear to constitute the natural termination of granular degeneration. Drowsiness and torpor are common symptoms throughout its course. Simple apoplexy, either suddenly supervening, or commencing in the form of stupor, which slowly advances to deep and imperturbable coma,—and without any commensurate or particular morbid appearance being presented within the head after death, is so frequent as to have universally attracted attention. When it presents itself in the advanced stage of granular degeneration, Dr. C. has generally observed it make its approaches gradually, first in the shape of unusual drowsiness and dimness of vision, then of constant torpidity, at length of stupor, which soon passes into complete and irrecoverable coma; and more than a week or ten days may elapse between the first indications and the final issue. In the early stage of the granular disease again,—and this secondary affection may occur in the very earliest stage,—its advances are more rapid: after a short preliminary stage of drowsiness, or delirium, or both together, deep stupor is quickly formed, and apoplectic coma supervenes; which may end fatally, sometimes without convulsions, more commonly with them, and in less than two days from the first appearance of head symptoms.

"Cases of this kind," he goes on to state, "are very generally connected with suppression of urine. Before the drowsiness or delirium comes on, the urine becomes greatly diminished in quantity, sometimes altogether suppressed; and the secretion very seldom continues a few days in this state without comatose symptoms beginning to threaten. In relation to this statement however two facts have struck me as not a little remarkable,—namely, that on the one hand extreme diminution of urine is not essential to the establishment of stupor and coma,—and that on the other hand stupor and fatal coma are not essential consequences of an excessive diminution. On the first point I may simply observe, that I have known coma form and prove speedily fatal, where thirty ounces of urine were discharged daily up to the time of death. As to the urine becoming much reduced in amount, and that for a great length of time, without coma necessarily following,—if we look merely to the solids of the urine as the essential object of this excretion, there can be no question that for weeks together the daily discharge may be reduced to one fourth of the natural amount, without any symptom of an affection of the head supervening. I have repeatedly witnessed such an occurrence; where moreover the analysis of the blood showed that it was loaded with urea. Farther, in one very interesting case, that of Johnston, No. 3, the patient passed no more than two ounces of light urine daily for nine days before death, yet he remained sensible to the very last minute of his existence, and died simply of inanition and exhastion from constant vomiting of every thing he swallowed. Still the reciprocal connexion between suppression of urine and coma as a general fact cannot be questioned. Whenever the urine continues very unusually reduced in amount, and still more altogether suppressed, the supervention of coma may commonly be looked for in the course of four days; and if drowsiness comes on, while the urine is in either of these states, fatal coma is seldom to be averted." 95.

Coma is not necessarily connected with dropsical effusions, for it may occur without any, or after they have been removed. But if the dropsical fluid be allowed greatly to accumulate, drowsiness, the first symptom of the affection of the head, very soon makes its appearance in the generality of cases; and it will speedily pass to fatal coma if not controlled. But the removal of the dropsy will usually remove the drowsiness.

Dr. Bright has seen the head affection assume the shape of epileptic convulsions.

Dr. Christison does not agree with Dr. Osborne in thinking that the comatose affection is usually a low form of arachnitis. It is always a very formidable symptom, and drowsiness, its herald, should be met with promptness.

"The connexion of coma and suppression of urine with granular disorganization of the kidnies is one of the most interesting facts which have been observed in its pathology. It advances us by one important step in our knowledge of suppression of urine considered as a disease. For I am persuaded, from what has been the result of the conjunct experience of the several practitioners of the Infirmary of Edinburgh for the last eight or ten years, that few cases of fatal suppression occur except in connexion with granular degeneration in its early or advanced stage; and in my own experience this connexion has been invariable. Several instances have presented themselves where the true origin of the disease was not suspected during life, but was discovered in the dead body." 97.

7. *Chronic Rheumatism.*

Dr. Christison regards this a frequent secondary affection. The form in which it commonly appears is that of mere neuralgia, without swelling or redness of the affected parts, and seated in the muscles more frequently than the joints: but sometimes swelling of the joints may be remarked.

8. *Pneumonia.*

Dr. C. has seen only two well-marked, and two latent cases of this kind.

9. *Diseased Heart.*

Dr. Christison is compelled to acknowledge the difficulty of determining, in many instances, which existed first, disease of the heart or of the kidneys. He observes:—

"The fact is that, with the exception of anasarca, and perhaps catarrh and dyspepsia, no complication is more common than that of granular disorganization of the kidnies with enlargement and obstruction of the heart, or with enlargement and tubercular derangement of the liver, or even with organic disease in both viscera at once. All who have written on the subject agree in this; and the ulterior experience of my colleagues and myself in the Edinburgh Infirmary completely corroborates what was stated to that purport by Dr. Bright, and afterwards in my own paper, as well as by Dr. Gregory. The exceeding frequency of disease of the heart in connexion with granular kidney and anasarca has even led some pathologists to doubt, whether the anasarca, which Dr. Bright and his followers have ascribed to the morbid condition of the latter organ, may not really arise from organic alteration in the former. And this doubt unquestionably derives some plausibility from the fact, that the respective state of advancement of disease in the two organs, equally with the history of the symptoms throughout their progress, indicates that of the heart to be often prior in its commencement. On the other hand however the same criterions will often show as clearly that the prior disease is the affection of the kidney; and besides there are very many cases where the affection of the kidney subsists, even in an advanced stage, without any change whatever in the structure of the heart." 100.

He goes on to add, that from the exceeding frequency of enlarged heart, with or without valvular obstruction, some conceive that its concurrence is more than accidental; and this suspicion derives support from the ascertained fact that in a large proportion of cases of such concurrence, the history of the symptoms, or the relative advancement of the two organic diseases as determined by inspection after death, shows that the disease of the kidney is the prior in date. If granular disorganization does really act as a predisposing cause in exciting enlargement of the heart, the probability is, as Dr. Bright points out, that this influence is exerted through the medium of the changes produced in the blood, which may act too powerfully as a stimulus to its contractions.

This is the point which offers the main obstacle to the reception, without considerable modifications, of the extreme views of Dr. Bright, Dr. Christison, and others. Those who receive those views with hesitation refer to the heart and liver as the frequent causes of the dropsy which the abovenamed gentlemen attribute to the kidney. And we must own that we think they often do so with justice. So far as we have seen, the heart and liver have played a far more prominent part in the production of dropsy than the kidneys have, and formed, after death, the more prominent lesions. The usual cause of diseased kidney is, à fortiori, a cause of diseased heart and liver—we mean, of course, intemperance. It is probable that the one does not so much produce the others, as that the same cause may give rise to all. The question is one of priority, and plus or minus. It is only by an accurate observation and record of facts that it can be solved.

### 10. *Diseased Liver.*

The difficulty of determining antecedence is admitted by Dr. Christison, even more explicitly in the instance of disease of the liver. The difficulty, too, of determining the alteration of that organ is insisted on. Percussion is a valuable adjuvant, but if there be only tubercular degeneration, without enlargement, percussion, of course, gives no assistance. The conjunction of ascites, to any *extent*, with anasarca, offers a means of diagnosis.

The connexion of the disease of the kidney and liver suggests the following train of doubts and of suggestions on the part of Dr. Christison.

"The conjunction of diseased liver, as well as of diseased heart, with granular degeneration of the kidnies is in all probability something more than accidental. The frequency of such conjunction is perhaps alone sufficient to show that they stand in some natural relation to one another. It is not improbable too that there is some alliance in nature between the morbid depositions on the valves of the heart, in the substance of the liver, and in the structure of the kidnies, by which the organic disease in each organ is essentially constituted. There is at least a resemblance in appearance and some similarity in consistence; but I am not aware that any attempt has yet been made to discover what analogy prevails between them in other respects, more especially in chemical properties and composition. In the meantime it seems tolerably well established, that the same constitutional state, whatsoever that state may be, which favours the development of granular disorganization of the kidnies, promotes also the formation of chronic organic disease in the liver, and of valvular obstructions of the heart.

These observations, on the mutual affinities between organic derangement of structure in the heart, liver, and kidnies, lead me to remark farther, that tubercular liver and depositions on the cardiac valves are not the only diseases in which granular disorganization of the kidnies appears in the shape of a secondary affection. It has been shown that many formidable diseases are apt to occur in constitutions invaded by the disorder of the kidnies. On the other hand this same disorder is apt to present itself in constitutions sapped by various other diseases. These it might be advantageous to inquire into in the present place. More facts however are still wanted to render the inquiry satisfactory." 105.

He adds that granular degeneration of the kidney appears to engender an infirmity of constitution which renders the body prone to diseases generally. This is shown by the frequency with which the kidneys are found more or less affected after death in a great variety of other disorders, although their condition may not have attracted notice during life, and although none of the more strictly secondary affections showed themselves. In particular it would appear that granular degeneration of the kidneys at a moderately advanced period of its progress renders the body peculiarly open to the invasion of some epidemic diseases. These organs have been frequently found far advanced in granular disorganization in cases of death from typhus, which for some years has been extensively epidemic in this city. And the same morbid appearance was found in a considerable proportion of the fatal cases of malignant cholera.

### IV. CAUSES.

Dr. Christison admits, what is more true than new, that the causes of most chronic visceral diseases are obscure. Nor is granular degeneration of the kidneys an exception.

The exciting cause is, very commonly, exposure to cold, or to cold and wet together. In four of M. Solon's cases, the patient referred his illness to a blow upon the loins. In many, if not in most instances, no obvious exciting cause is recognizable.

It is probable that some constitutional infirmity must have predisposed to the compliant. Yet such infirmity is not always evident, and the apparently robust are victims to it. But, of all the predisposing causes, none has appeared to play so important a part in Edinburgh, as intemperance. A large proportion of cases have occurred in the persons of habitual drunkards. It is not necessary however that the vice of intemperance should be carried to so great an excess; for a still larger proportion perhaps is composed of those, who, without deserving the designation of habitual drunkards, are in the constant practice of using ardent spirits several times in the course of the day, and of occasionally indulging to intoxication. Dr. C. thinks that he is justified in reckoning the proportion of cases referable to intemperance at three-fourths, or even four-fifths of the whole. Nor is there any difficulty in conceiving that the stimulation of the kidney by fermented liquors should induce disease of it.

But, as Dr. Christison observes, granular degeneration may occur in persons who have not been intemperate. It is occasionally noticed at a period of life when habits of intemperance are out of the question.

"In all such cases, and I may add in all other instances where the disease has appeared either during adolescence or in young adults, whether connected or not with intemperate habits, the individuals have appeared to me to present characteristically the peculiarities of the strumous constitution. The same connexion may be often traced in persons of middle age or advanced life by mere inspection of their physical development; and in others, where the physical characters are obscure, the relationship may still be discovered by the presence of other strumous disorders, or a liability to them,—as for example by indolent chronic ulcers of the legs, scars indicating their occurrence at former periods, or tendency in youth to enlargement of the lymphatic glands. I have very little hesitation therefore in putting down the scrofulous diathesis among the predisposing causes of granular disorganization of the kidnies. The connexion has even at times seemed so invariable, that I have been inclined to suspect the strumous diathesis to be the prime and only essential condition, and intemperance no more than an accessary predisposing cause in any case. However this may be, there seems little doubt, that the disease is in no circumstances developed with greater certainty than where both conditions concur,—where habits of intemperance have been engrafted upon a strumous taint of the constitution." 112.

Dr. Christison seems struck with the idea of a connexion between scrofula and granular degeneration of the kidney. He almost hints that the latter will turn out a result of the former. He leans aslant upon the occasional connexion of phthisis and granular degeneration, a connexion which we must confess we think unfrequent.

Scarlatina is another predisposing, perhaps exciting cause. Dr. Christison reluctantly owns that it may admit of question whether all cases of dropsy, after scarlatina, are connected with granular degeneration of the kidneys. But he eagerly affirms that such is the nature of a very large proportion of them.

After again owning the meagre nature of our information, Dr. Christison adds:—

"Several agents were formerly mentioned, namely, mercury, cantharides, and peculiar articles of diet, which have the property of inducing an albuminous impregnation of the urine in particular individuals. These facts were adverted to as a caution against the unreserved conclusion that albuminous urine alone will infer the presence of kidney disease. But as the foreign impregnation arises from the causes in question only in some individuals,—as instances of the kind are indeed rather uncommon,—it may fairly be made the subject of inquiry, whether they do not present themselves in those only who are by constitution predisposed to granular kidney, and consequently whether they may not be considered as instances, where the disease is threatened, and might be induced by frequent repetition of the exciting agent. I think it deserves inquiry whether the use or abuse of mercury in particular constitutions may not lead to granular disorganization of the kidnies: for I have met with a sufficient number of cases to excite a suspicion to this effect." 116.

Dr. Christison's suspicion may prove accurate. Yet it must be obvious how loth he is to connect albuminous urine with any thing but granular degeneration of the kidney.

The complaint is more frequent, and the reason why it should be so is obvious, in males than females—at middle life than at any other period. It is most rife between the ages of thirty and fifty. But old age is not exempt from it. Our author had lately under his care a man of seventy-nine, who was affected with it in the middle stage, and who recovered both from extensive dropsy and from long-continued obstinate diarrhœa; and at the age of sixty it is not uncommon. On the other hand it is often enough met with in early adolescence. He has twice had fatal cases at the age of seven or eight years; in another instance at the same age complete recovery was accomplished; and if we are to suppose, he says, that all cases of inflammatory dropsy after scarlatina depend on granular disease of the kidneys, which may be strongly suspected to be the fact, then the occurrence of this disease in infancy must be considered as a familiar event.

The disease *seems* less frequent in the middle than in the lower classes. Likely enough; for the former are probably less intemperate, and certainly less exposed to wet and cold.

Dr. Christison contributes some scanty observations to the elucidation of the influence of modes of occupation on the production, or on the course of the disease. Among twenty-six males, there were three night-watchmen, three dissipated old soldiers, three weavers, two labourers, two street-porters, a blacksmith, a distiller, a town-carter, a country surgeon, two seamen, a travelling hawker, a mason,—twenty-one in all, whose occupations subjected them either to unusual exposure or to intemperance, or to both together.

### V. PROGNOSIS.

1. This forms the subject of the fifth section. Dr. Christison, upon the whole, does not take so gloomy a view of the disease as might be thought. And he is right, for our knowledge is too recent and too vague to permit us to say positively what lesion may and what may not be recovered from. Where the disease has been recent, it has seemed entirely removed. Recovery is not uncommon, from the dropsy succeeding scarlatina. In other cases every symptom has disappeared except coagulability of the urine, and the individuals have continued for a long time afterwards to follow a laborious occupation in the enjoyment of tolerable health and without any material uneasiness. But we need not add that, so long as albumen is secreted by the kidneys, the case must be regarded with distrust.

"That in its advanced stage, when the cortical and tubular textures of the kidnies have been invaded and partly destroyed, the disease must ever remain incurable, I need hardly say. For even though the granular deposit should be absorbed and thrown off,—of the possibility of which, moreover, there is hitherto no clear evidence, still medical art cannot be expected to restore what has been lost of the specific renal structure. But I cannot help thinking that in such a situation some physicians are prone to entertain an unnecessarily desponding view of their patient's predicament. It has seemed to me clear, that, even where the disease has reached a pretty advanced stage in its progress, the patient may be brought by due treatment into such a condition, as to live without material uneasiness for a term of years, of which our present knowledge cannot well settle the limit;—provided he avoid improper exposure to cold, intemperance, and other irregularities in regimen. It is impossible perhaps to remove the disorganization: but its farther advancement may be checked for a time, before it has proceeded so far as to be incompatible with the enjoyment of tolerable health and comfort. Such in particular is not unfrequently the result where no other secondary disorder has fastened on the constitution except dropsy." 123.

2. Dr. Christison makes some remarks on the prognosis of the several secondary affections. The only one which we need notice, refers to the removal of the dropsy from diseased kidneys. This is usually, though gradually, effected.

3. The special indications of a favourable or unfavourable issue from the conclusion of this section.

The risk to life, writes Dr. Christison, is by no means proportioned to the amount of albumen in the urine. The reverse indeed holds true in some measure. For where the albumen abounds, the organic derangement of structure is commonly in its early stage; and hence there is less immediate danger to life, provided the concurring incidental diseases, which in this stage are chiefly inflammatory in their nature, be actively treated. The diminution and ultimate disappearance of the albumen from the urine is a favourable sign, and is generally accompanied with marked amendment in other more tangible points. Singly however it may be a doubtful prognostic. If attended with a moderately high or gradually increasing density of the urine, whether with or without an increase in its quantity, the diminution and disappearance of albumen are favourable signs. But this will, on the contrary, rather indicate a gradual advancement of the disease, if the density of the urine should at the same time slowly decrease, especially where its quantity remains stationary. Diminution of the albumen, with increased quantity and diminished density, cannot be relied on as a prognostic on either side.

The risk, he goes on to add, is not altogether proportional, as might reasonably be supposed, to the inflammatory condition of the blood. The blood seldom presents the buffy coat so well-marked as at the commencement of the organic disease in the kidneys. Such a state of the blood clearly indicates, it is true, a greater risk of incidental local inflammations arising. But then it is, in common with such inflammations, under the control of medical art.—The danger to life may be considered material in all circumstances where the blood is greatly defective in colouring matter, provided the diminution be the result of the disease, and not of other incidental causes, such as frequent blood-letting. The decrease in the proportion of colouring matter may be held, as was formerly explained, to be a correct measure of the progress made by the disorganizing agent. It need scarcely be added that the disappearance of an inflammatory state of the blood is a favourable sign, because there is less risk of the supervention of local inflammation.

We need scarcely observe, after what has been already said, that the risk of life is not necessarily in proportion to the amount of dropsical effusion, for this is generally within the range of remedies. But though the disappearance of dropsy is a favourable sign, yet it does not necessarily indicate the removal of the tendency to other secondary affections; nay, coma does occasionally follow hard upon it.

"The patient's danger is on the whole in proportion to the lowness of the density of the urine; and the reason obviously is, that the lower the density of the urine, the farther has the organic alteration in the structure of the kidney advanced in its progress. This rule however applies only where the quantity of the urine is not materially greater than the natural average. On the other hand, it applies with peculiar force where such urine is also defective in quantity. For example, the patient may always be considered in imminent danger where the urine has a density of 1008 or 1010, and its quantity does not exceed twelve ounces daily.

In reference to this article of the prognosis, it would perhaps be preferable that physicians were to look less to the mere density and quantity of the urine abstractly, and more to these qualities as constituting a measure of the amount of daily solids excreted. It is the diminution in the daily discharge of solids with the urine that constitutes essentially the unfavourable prognostic. Nature allows of a considerable variety in respect of the discharge of solids in the urine without the health being necessarily affected. This may be remarked on studying the condition of the urine both in different individuals and in the same individual at different times. The history of the present disease shows that a very extraordinary diminution from the natural standard may take place for a great length of time, without at all events any immediate or very obvious risk to life. We see patients frequently living for many weeks in the comfortable enjoyment of tolerable health, though the amount of solid excretion by urine is diminished to fully one-third of the natural daily discharge. Seldom however does the quantity fall to one-fourth without troublesome secondary disorders forming; and any material reduction under that amount is speedily followed by urgent symptoms, most generally by drowsiness, leading on to stupor and coma." 131.

Suppression of urine is a very fatal symptom. Dr. C. has never known (save once) a patient survive more than a few days, when the daily quantity of urine has fallen even to three or four ounces. In that case, however, of suppression with acute dropsy, the patient recovered under active antiphlogistic treatment.

Gradual augmentation of the density of the urine, the quantity at the same time being natural, or at least not much below the natural standard, is always a propitious symptom.

### VI. Treatment.

This, as usual, winds up the descriptive and doctrinal part of the work.

It need hardly be observed, that the treatment resolves itself into that required for the primary, and that applicable to the secondary affections.

1. *Treatment of the Primary Disease.*—In the early stage, the symptoms usually indicate, and experience confirms, the utility of active antiphlogistic treatment, of venæsection, followed by leeches and cupping on the loins, and these succeeded by blisters, setons, or issues, as the case may be. Dr. Christison recommends as an index of the propriety of following up depletion, a careful examination of the composition of the blood, more particularly in reference to the amount of its hæmatosin. The best sign of improvement from depletion is when the urine speedily increases in quantity, loses more or less of its coagulability, and maintains or increases its density.

Dr. Christison passes to diaphoretics, so naturally and so much recommended, especially by Dr. Oborne. Yet Dr. Christison hints exaggeration in the confident and flattering statements of the latter, and we cannot but think that he *has* outrun the anticipations which experience sanctions.

"I sincerely wish," says Dr. Christison, "that my experience of the effects of the diaphoretic plan could bear out the very sanguine encomiums bestowed upon it by Dr. Osborne. He endeavours to show by a numerical statement, that his success with diaphoretics has greatly exceeded that of any other practitioner who has followed a different method of cure. He alleges in regard to dropsy, that he ' never failed in removing it whenever the entire surface was restored to a perspiring state.' And in another passage he writes in Italics that ' whenever general perspiration came on, either spontaneously or in consequence of medicine, then the cases always terminated favourably.' Since the announcement of his method I have often resorted to it, sometimes with evident advantage, much more generally without success; and I must likewise add, that I have several times seen general perspiration, both spontaneous, and from the use of diaphoretics, fail to produce any material relief. Still no one can question the general propriety of the diaphoretic method of cure." 137.

The most efficient diaphoretic, in our author's hands, has been Dover's powder in the dose of five or eight grains three times a-day. With this should always be conjoined the warm-bath every twenty-four or forty-eight hours. No other remedy gives more relief for a time, and patients who have had it once, in general importune the physician to have it repeated ; so that its ultimate good effects can scarcely be doubted. It commonly occasions sweating for some time afterwards, and is followed by quiet sleep. Extract of hyoscyamus is a good sedative—James's powder another eligible diaphoretic.

Laxatives are useful, active purgatives hazardous, save when dropsy or coma supervenes. Dr. Christison speaks rather favourably than otherwise of diuretics. After noticing the objections which have been advanced against them, objections both of fact and theory, Dr. C. observes:—

" Here," however, " it appears to me that the argument has been pushed too far, and to the establishment of a serious practical error. It does not follow, because diuretics by their stimulus cause increased flow of natural urine, that they will also cause an increase of morbid secretion. The irritation which excites the former may be different from that which excites the latter. That they really are different in kind would appear probable, as well from the extreme difference of their products as from the fact that diuretics, when they increase the flow of urine in this disease, very rarely, so far as I have observed, increase the albumen, which in the early stage may be held to be a correct measure of the degree of morbid irritation. I have even repeatedly seen the albumen disappear under diuretics. But, if the two irritations be different in kind, we may infer from numberless parallel instances in regard to inflammation and irritation in the organs and textures of the body at large, that the one may be induced, without necessarily increasing, nay possibly enough with the effect of diminishing, the other. Theory therefore is not at variance, as some imagine, with the employment of diuretics in granular disease of the kidnies. Neither for my own part have I had occasion to observe any distinct facts which would lead to a conclusion in any way different. Diuretics, I repeat, do not increase the coagulability of the urine in the early stage: In many instances they seem to diminish it. In the advanced stage there are no easy and sure criterions for judging of the progress of the primary disease ; but so far as one may judge, it does not appear that disorganization at this period is promoted by the operation of diuretics." 140.

So far as we have seen, diuretics have not appeared to benefit, but rather to aggravate the renal symptoms. When irritation of the kidney has been conjoined with an albuminous condition of the urine we are satisfied that, in our hands, diuretics have *not* proved of service. When dropsy or coma has supervened or is impending, the case, no doubt, is altered. Yet, even here, we conceive that caution should be used, and that the possibility of acting injuriously on the kidney should be kept in view.

We are not quite converts to Dr. Christison's argument. What he says may be very true, but it does not seem to us consistent with either analogy or probability. If a man has an inflamed eye, exposure of it to light, or exertion of it in vision is not found to be beneficial to it. If he has inflamed stomach, a hearty meal is rather apt to disagree. If muco-enteritis obtains, drastic purgatives are not quite the thing; and inflammation of the knee-joint is seldom much the better for a run or a galopade. Whichever way we turn we find that when an organ is inflamed the stimulating it to its usual actions tends to increase the excitement and the inflammation, and repose is an essential item of treatment. Why a kidney in an inflammatory state, should be benefitted by being set to additional work, we are unable, on the grounds of reason, to perceive.

Dr. Christison apologises hesitatingly for mercury. On the whole he agrees with those who denounce it. Yet he owns to its employment in small doses in aiding the action of diuretics and cathartics, nay he professes that a sore mouth so produced has not seemed to him so great an evil as it has been thought by others. Our prepossessions are against mercury, and we rather agree with those who are inclined to prohibit its use, than with those who resort to it, though cautiously and timidly.

When granular disorganization of the kidneys has reached the middle or advanced stage, or where it has been chronic throughout, there is little room for active treatment. Yet even in this condition, much may be done for the improvement of health, and the prolongation of life. A rather advanced state of disorganization is not incompatible with the enjoyment of tolerable health and comfort. For this end, Dr. Christison observes, a provision seems to be frequently made by a spontaneous increase in the quantity of urine; so that the daily amount of solid secretion through that channel is maintained about the natural standard. Yet such provision is not indispensable; for cases are observed where health and general comfort are preserved for a considerable length of time, although the diminished density of the urine is not compensated by any material increase in quantity.

The treatment adapted to the arrest of the progress of the disorganization or its mitigation, consists mainly in dietetic and general measures. The maintenance of the cutaneous transpiration by warm clothing, the avoidance of alternations of temperature, the occasional employment of the warm-bath, and the enforcement of regular active exercise, are the most important articles of the treatment; and next may be mentioned regularity and moderation as to food and drink, which are to be secured by a reduction of the amount of food if the patient belong to the middle ranks of life, by the selection of such articles as are both nutritive and easily digestible, probably by observing a preponderance of vegetable food, and certainly by entire abstinence from spirituous liquors as well as the sparing use of wine. Dr. Christison makes no mention of sarsaparilla. Yet we have in several instances conceived that, after inflammatory action was subdued, this, as well as several light bitters, combined with henbane, and, if the urine was acid, with soda, have proved of service.

*Treatment of the Secondary Affections.*

The treatment requisite for these affections, occurring as primary disorders, requires some modification in this their secondary shape. Dr. Christison dwells on the admitted fact, that all are exceedingly apt to arise under exposure to cold, producing in all probability impaired transpiration; and consequently that in most of them advantage will be found in keeping diaphoretics as much in view as possible in the course of the treatment.

1. *Anasarca.*—When this appears in the acute form, as is usually the case when it occurs in the early stage of the disease, free blood-letting must be resorted to. The correct guides for regulating it are the state of the pulse, the local pains, the breathing, the temperature of the skin, and the general feelings of the patient. The presence of the buffy coat cannot be trusted to as an index of the propriety of further depletion; for a highly buffy state of the blood often occurs where no particular excitement prevails, or may continue after excitement is effectually subdued. When blood-letting is found necessary for acute dropsy in the advanced stage of the primary disease, it must be employed with a more sparing hand.

When the general excitement has been put down by blood-letting, or when there is not enough to require it, the choice of remedies lies between diaphoretics, diuretics, and purgatives. Dr. Christison, as has been observed, doubts the justice of the current encomia on diaphoretics. He leans on diuretics, and amongst these he singles out digitalis and cream of tartar, as the objects of his preference.

"No combination which I have tried combines both advantages in such a degree as digitalis and cream of tartar together. The former was usually given in the dose of one or two grains of the powder in the form of pill thrice a-day, or in the dose of ten, fifteen, or twenty minims of the tincture three times daily in a little distilled water of cinnamon or cassia. The cream of tartar was administered thrice a day in the quantity of a drachm and a-half or two drachms with about five ounces of water. Diuresis may generally be induced by such means in the course of three or four days, sometimes sooner,—seldom, however, if delayed beyond the seventh day. I cannot comprehend why it should be said by some that this method seldom succeeds at all in exciting diuresis. Occasionally it appears to be promoted by giving also a mercurial pill every evening for four or five days, which may be omitted soon after the flow of urine increases, in order to avoid the risk already adverted to of the constitution being extremely sensible to mercurial action. Where diuretics had been given for some time without effect, I have sometimes seen their action suddenly developed by the administration of an emetic of tartar-emetic and ipecacuan. Still more frequently have I seen it brought on in the same circumstances by a single dose of some hydragogue cathartic, such as gamboge. If all these means fail, and it should still be thought advisable to persevere with the diuretic method, the powder of squill may be tried, or infusion of broom-tops, or spirit of nitric ether, or hollands with water, or carbonate, nitrate, or acetate of potash." 150.

If the cream of tartar acts excessively as a hydragogue cathartic, that operation must be controlled by opium. The removal of the dropsy by diuresis is often slow, less so, perhaps, in the advanced than in the early stage.

Purgatives are not, at present, quite so much in vogue as formerly. But Dr. Christison evinces some partiality for gamboge.

"I have often observed this particular remedy,—which it is the fashion to disregard and decry at present, because it constitutes the greater part of a much vaunted and notoriously unsafe nostrum,—to act with great force both in occasioning free watery evacuations and in reducing the dropsy, yet without any particular tormina, exhaustion, or other uneasiness being occasioned, although it was administered once every two days, or even daily. As to the special objections to the employment of purgatives in granular disease of the kidnies, they are clearly inadmissible where an obstinate exhausting diarrhœa is present; and I must likewise allow that I have occasionally seen them apparently succeeded by this affection." 152.

And he observes farther on :—

"Gamboge is the purgative I have most frequently employed, and generally in the dose of five grains, sometimes seven, very rarely nine. It appears of consequence to secure its being very finely divided by carefully pulverizing it with half a drachm of bitartrate of potash, otherwise it is more apt to excite griping. The rough-looking gamboge of Ceylon has seemed not less efficacious and convenient than the finest Siam varieties; and I have no doubt the one may be substituted for the other for all medicinal purposes. It should be given once every two days, except in urgent circumstances, or where a trial of it daily has shown it may be advantageously and conveniently given so often. I have occasionally employed elaterium for the same purposes and also with good effect, in the dose of a quarter of a grain. Sometimes bitartrate of potash alone acts powerfully as a hydragogue cathartic in the dropsy which accompanies granular disorganization of the kidnies. It has been already mentioned that when given in two-drachm doses, with the view of exciting a flow of urine, the bowels are sometimes acted on profusely instead of the kidnies; and this action may be induced with tolerable certainty by daily doses of half an ounce. But on the whole gamboge is the hydragogue which has appeared the most certain and most easily managed." 154.

We may remark that Dr. Christison scarcely does justice to the elaterium. English physicians give their suffrages to this powerful medicine more liberally, and, we conceive, with justice.

The anasarca may extend to such a degree as to demand punctures. There cannot be a doubt of the superiority of needle punctures over scarifications. Yet we agree with Dr. Christison in the statement that the former are not so absolutely free from danger as some, who recommend them, would wish us to believe. He has seen two fatal cases of sloughing after them, and we have met with one. A minor degree of erythema is common.

Diaphoretics should be conjoined with the purgative and diuretic plans in the treatment of anasarca; and they must be continued vigorously long after the dropsical effusion is evacuated.

The purgative plan is probably better adapted to the early than to the advanced stage of granular kidney. In the latter stage troublesome diarrhœa is much more frequent than in the former.

On the treatment of *Dyspepsia*, of *Diarrhœa*, of *Inflammation of the Serous Membranes*, and of *Catarrh*, Dr. Christison says nothing to detain us.

When *coma* occurs in the early stage of the primary disease, bloodletting, purgatives, and diuretics are the remedies. When it occurs in the advanced stage, moderate evacuations may be employed, but will probably fail.

We have now presented, we may say, the work of Dr. Christison, rather than a notice of it. We shall pursue the subject of Urinary Diseases in our next number.

